# Evolutionary relationships and taxonomy of *Microtea* (Microteaceae), a basal lineage in the core Caryophyllales

**DOI:** 10.3897/phytokeys.115.29041

**Published:** 2019-01-09

**Authors:** Alexander P. Sukhorukov, Alexander N. Sennikov, Maya V. Nilova, Yuri Mazei, Maria Kushunina, Maria Salete Marchioretto, Pavel Hanáček

**Affiliations:** 1 Department of Higher Plants, Biological Faculty, Lomonosov Moscow State University, 119234, Moscow, Russia; 2 Botanical Museum, Finnish Museum of Natural History, P.O. Box 7, 00014 University of Helsinki, Finland; 3 Herbarium, Komarov Botanical Institute of Russian Academy of Sciences, Prof. Popov St. 2, 197376 St. Petersburg, Russia; 4 Department of Hydrobiology, Biological Faculty, Lomonosov Moscow State University, 119234, Moscow, Russia; 5 Department of Plant Physiology, Biological Faculty, Lomonosov Moscow State University, 119234, Moscow, Russia; 6 Instituto Anchietano de Pesquisas/UNISINOS, São Leopoldo, RS, Brazil; 7 Department of Plant Biology, Mendel University in Brno, Zemědělská 1, 613 00 Brno, Czech Republic

**Keywords:** Caryophyllales, *
Microtea
*, molecular phylogeny, reproductive characters, taxonomy

## Abstract

The basal position of the small American genus *Microtea* within the core Caryophyllales was suggested only recently in accordance with molecular phylogeny. However, the specific relationships within the genus were not traced. The results of our phylogenetic analysis based on the *mat*K chloroplast gene suggest the monophyly of *Microtea*, and *Ancistrocarpus* and other related genera should be included in it. *Microtea* is divided into two major sister clades: clade A consisting of *M.glochidiata*, *M.maypurensis* and *M.tenuifolia*, and clade B comprising *M.debilis*, *M.sulcicaulis, M.scabrida*, *M.celosioides*, and *M.papillosa*. The nrDNA dataset (ITS), although containing only a limited number of accessions, shows the same species number in clade A, and the remaining species studied (*M.debilis*, *M.scabrida* and *M.celosioides*) form clade B. Subgeneric status is assigned to clades A and B corresponding with the names Microteasubgen.Ancistrocarpus subgen. nov. and Microteasubgen.Microtea, respectively. The diagnostic characters at the subgeneric level are as follows: length of pedicels, number of flowers at each node, number of stamens and styles. A multivariate analysis of 13 distinguishing morphological characters supports the results of phylogenetic analysis. All species have similar pericarp and seed ultrasculpture and anatomy, and they share the reticulate pericarp surface (independent of presence or absence of finger-shaped outgrowths on its surface) and rugose or slightly alveolate seed ultrasculpture. On the basis of morphological characters, we accept 10 *Microtea* species. A checklist includes a new diagnostic key, morphological descriptions and distribution patterns of each species. *Galeniacelosioides* is the oldest legitimate name available for the plants previously known as *Microteapaniculata*, for which the combination *Microteacelosioides* is validated here. The neotypes of *Galeniacelosioides* and *Microteasprengelii* were designated from the collections of Prinz Wied at BR. The name *M.foliosa* is discussed and finally synonymized with *M.scabrida*. The lectotypes of *Ancistrocarpusmaypurensis* (≡*Microteamaypurensis*), Microteadebilisvar.ovata (=*M.debilis*), *M.glochidiata*, M.maypurensisvar.angustifolia (=*M.tenuifolia*), M.glochidiataf.lanceolata (=*M.maypurensis*), *M.longebracteata* (=*M.celosioides*), M.paniculatavar.latifolia (=*M.scabrida*), *M.portoricensis*, *M.scabrida*, *M.sulcicaulis*, and *Potamophilaparviflora* (=*M.maypurensis*) are designated. *Microteasulcicaulis* is reported for the first time as native to Bolivia, and *M.maypurensis* is reported from Indonesia (Java), where it is found as an alien plant with an unclear invasion status.

## Introduction

The genus *Microtea* Sw. was described by Swartz (1788) with one species, *M.debilis* Sw., native to the Lesser Antilles (the Caribbean). The author placed it within the group “Pentandria–Digynia” due to the pentaphyllous perianth, five stamens, and two styles. A pericarp with distinct echinate outgrowths was reported as another indicative character of *Microtea* (Swartz 1788). Kunth (1817) described a related genus *Ancistrocarpus* Kunth with the type species *A.maypurensis* Kunth, and he pointed out that the main differences between *Microtea* and *Ancistrocarpus* belong to the reproductive characters such as the different numbers of stamens (five vs eight, respectively) and styles (two vs four or five) and the shape of the pericarp outgrowths (echinate vs apically hooked). Further new generic and species names were mostly based on the same reproductive characters (Roemer and Schultes 1820, Schrank 1821, Link 1821), but none of the genera allied to *Microtea* have been commonly accepted, and currently *Microtea* has been considered a single genus that includes all closely related taxa (e.g., Steudel 1841, Moquin-Tandon 1849, Urban 1885, Walter 1909, Rohwer 1993, Marchioretto and de Siqueira 1998). According to the latest studies (Marchioretto and de Siqueira 1998, Hernández-Ledesma et al. 2015), *Microtea* comprises 10–12 species distributed in Central and South America. They can be distinguished and classified by life history, presence of bracteoles by each flower, and morphology of the pericarp (e.g., Walter 1909, Marchioretto and de Siqueira 1998). However, many important reproductive traits are still poorly studied in this genus, including the fruit and seed anatomy that has been depicted schematically only for *M.debilis* (Melikian 1993).

Traditionally, *Microtea* occupied a provisional position within the core Caryophyllales and has been considered as part of the Chenopodiaceae (Kunth 1817, Walter 1906, Takhtajan 2009), Petiveriaceae (Brown and Varadarajan 1985) or Phytolaccaceae (Moquin-Tandon 1849, Walter 1909, Hutchinson 1926, Nowicke 1968, Behnke 1993, Rohwer 1993, Atha 2004, Zhu and Sanderson 2017). Friedrich (1956) suggested that *Microtea* may be a connecting link between the Phytolaccaceae and Chenopodiaceae. Also, Behnke (1993) and Behnke and Mabry (1994) reported that the structures of sieve-element plastids and of the pollen grains deviate from those of the other Phytolaccaceae. The recently combined molecular phylogeny based on the *petD* and *matK* regions revealed a distant position of *Microtea* from both Chenopodiaceae and Phytolaccaceae (Schäferhoff et al. 2009). However, only two *Microtea* species – *M.debilis* and *M.scabrida* Urb. – were included in this molecular analysis (Schäferhoff et al. 2009). Currently, the monophyly of *Microtea* and the relationships between its species have not been confirmed.

The aims of the present paper are (1) to include more species of *Microtea* in the molecular analysis in order to clarify the relationships between the species of the genus and to confirm the monophyly of *Microtea*, (2) to provide new data on the carpological characters as the most diverse and taxonomically important traits, and (3) to provide a new taxonomic description of the genus and better determination of the range of each species.

## Methods

### Field studies and revision of the herbarium material

Field work was done by the first author (AS) in Grenada in November 2016 (Main Island, Carriacou, and Petit Martinique) and in March 2018 in the Dutch Caribbean (Curaçao); however, no *Microtea* species were found. The field investigations in Brazil were provided by Maria Salete Marchioretto. The revision of herbarium specimens was undertaken in B, BM, BR, E, G, H, K, L (incl. U & WAG), LE, LY, M, MEXU, MHA, MSB, MW, P, and PACA. The Virtual database of the Brazilian herbaria (http://reflora.jbrj.gov.br/reflora/PrincipalUC/PrincipalUC.do), National Herbarium of Colombia (http://ciencias.bogota.unal.edu.co/icn/colecciones-cientificas/herbario/) and the Tropicos database (http://tropicos.org/Name/24800059?tab=specimens) were used as references for some specimens kept in ASE, CEN, COL, FURB, GB, HUFS, NY, NYBG, RB, SJRP, and US if their identification was possible using the digital images.

### Carpological studies

Several fruits of all species were taken from the herbaria vouchers deposited in herbaria with the permission of the curators. Seed ornamentation was examined using a scanning electron microscope (SEM) JSM–6380 (JEOL Ltd., Japan) at 15 kV after sputter coating with gold-palladium in the laboratory of Electron Microscopy at the Lomonosov Moscow State University. To restore the soft pericarp tissue prior to scanning electron microscopy, the fruits were dehydrated in aqueous ethyl alcohol solutions of increasing concentration, followed by alcohol-acetone solutions and pure acetone. The seeds did not require a complicated treatment prior to SEM due to the presence of the hard seed coat. The cross-sections of the fruits and seeds were prepared using a rotary microtome Microm HM 355S (Thermo Fisher Scientific, USA). Before sectioning, the seeds were soaked in water:alcohol:glycerin (1:1:1) solution, dehydrated in an ethanol dilution series and embedded in Technovit 7100 resin (Heraeus Kulzer, Germany). The cross-sections were observed using a Nikon Eclipse Ci microscope and photographed with a Nikon DS-Vi1 camera (Nikon Corporation, Japan) at the Department of Higher Plants (Moscow State University).

### Phylogenetic analysis

The list of vouchers and their accession numbers is provided in Table 1.

**Table 1.** Voucher information and GenBank accession numbers for *Microtea* species and outgroups included in the phylogenetic analysis. Sample codes are provided only for the newly sequenced samples.

**Sample code**	**Species**	**Voucher**	**GenBank accession number**	
			***mat*K**	**ITS**
Mi02	* Microtea debilis *	French Guiana, Eau Claire, 15 Aug 1993, *S. Mori et al. 23295* (P05197089)	MH678599	–
–	* M. debilis *	USA, California, cultivated, *Yuncker et al.* (UC851834)	–	JX232577
Mi14	* M. celosioides *	Brazil, Bahia, Milagres, 6 Mar 1977, *R.M. Harley 19451* (U 1473444)	MH678600	MH726167
Mi07	* M. celosioides *	Brazil, Piaui, Caracol, 25 Feb 2011, *Melo et al. 9216* (PACA 115982, sub *M.longebracteata*)	MH678601	–
Mi06	* M. glochidiata *	Brazil, Tucano Mun., 20 Feb 1992, *A.M. de Carvalho & D.J.N. Hind 3841* (G)	MH678602	MH726168
Mi10	* M. maypurensis *	Brazil, Bahia, Rio Jacurici, 16 Jan 1997, *M.M. Arbo et al. 7276* (G)	MH678603	MH726169
Mi22	* M. papillosa *	Brazil, Minas Gerais, Diamantina, 11 May 1982, *L. Rossi et al. 76279* (PACA 76279)	MH678604	–
Mi19	* M. scabrida *	Paraguay, Concepcion, Paso Horqueta, 18 Nov 1993, *E. Zardini & T. Tilleria 37460* (MW0581802)	MH678605	–
Mi26	* M. scabrida *	Paraguay, Cordillera Dept., Cerro Tobati, 28 Oct1987, *R. Degun & E. Zardini 447* (G)	MH678606	MH726170
Mi27	* M. scabrida *	Paraguay, Guaira, Melgarejo, 13 Mar 1989, *E. Zardini & C. Velasquez 11391* (MW)	MH678607	MH726171
Mi28	* M. scabrida *	Paraguay, National Park Ybicu’i, 11 Nov 1989, *E. Zardini & U. Velásquez s.n.* (G)	MH678608	–
Mi47	* M. sulcicaulis *	Paraguay, dept. Cordillera, Colonia Rosado, 26 Oct 1986, *A. Schinini & E. Bordas 24850* (G)	MH678609	–
Mi34	* M. tenuifolia *	Brazil, Bahia, 23 Mar 1974, *Belmonte 17305* (U1473428)	MH678610	MH726172
Mi36	* Griselinia littoralis *	Switzerland, Botanical Garden of Geneva, Sep 2017, *A. Konstantinova* (living collection)	MH678611	MH726173
Mi41	* G. scandens *	Chile, Santiago, 1925, *A. Marillo 923* (LE)	MH678612	MH726174
–	* Macarthuria australis *	Australia, *Lepschi & Brims 1943* (K)	FN825765	–
–	* M. neocambrica *	Australia, *Coveny & Wilson 11674* (K)	FN825766	–
–	* Stegnosperma halimifolium *	no data	HQ878442	–
–	* Simmondsia chinensis *	no data	AF204863	–

### *DNA extraction and PCR amplification*

The nuclear (ITS) and chloroplast (*mat*K) regions of genomic DNA were used for the phylogenetic analysis. Total DNA was isolated from dried leaves using Invisorb® Spin Plant Mini Kit (Stratec Molecular GmbH, Berlin, Germany). ITS-A (Blattner and Kadereit 1999) and ITS4 (White et al. 1990) primers were used for ITS region amplification, and MatK-1RKIM-f and MatK-3FKIM-r were used for *mat*K region (http://botany.si.edu/projects/dnabarcode/matK_PCR_&_Sequencing_Protocols.pdf). PCRs were performed using 0.75 units of MyTaq Red DNA polymerase (Bioline, London, UK) in 15 μl of original buffer containing MgCl_2_ and dNTPs, with 0.3 μM of each primer and 1 μl of unquantified DNA template. Thermocycling was carried out in TProfessional Basic Thermocycler (Biometra, Göttingen, Germany) using the thermal and cycling conditions as described in Shaw et al. (2007): initial denaturation at 80 °C for 5 min; 30 cycles of 95 °C for 1 min, 50 °C for 1 min, a ramp of 0.3 °C/s to 65 °C and incubation at 65 °C for 4 min, with a final extension step of 65 °C for 5 min. A clean-up reaction with exonuclease I and alkaline phosphatase (Thermo Fisher Scientific, Waltham, Massachusetts, USA) was used to remove unincorporated primers and nucleotides before sequencing. The PCR products were sent to Macrogen Europe (Netherlands) for automated sequencing. The primers used for amplification were also used for the sequencing reactions.

### Sequence alignment and phylogenetic reconstruction

Data files were assembled, edited and evaluated using Geneious 8.1 software (Biomatters Ltd, Auckland, New Zealand). Regions of ambiguous alignment were excluded from all analyses. After exclusion of these regions we used 754 characters in the nuclear (ITS) and 828 characters in the chloroplast (*mat*K) analysis. The nuclear and chloroplast data were analyzed separately with MEGA7 software (Kumar et al. 2016) which delivered a maximum likelihood (ML) tree based on the Kimura 2-parameter model (Kimura 1980) with support for nodes measured by bootstrap percentages (N70% considered significant). The percentage of trees in which the associated taxa clustered together is shown next to the branches. Initial tree(s) for the heuristic search were obtained automatically by applying Neighbor-Join and BioNJ algorithms to a matrix of pairwise distances estimated using the Maximum Composite Likelihood (MCL) approach, and then selecting the topology with superior log likelihood value. The tree is drawn to scale, with branch lengths measured in the number of substitutions per site. Evolutionary analyses were conducted in MEGA7 (Kumar et al. 2016).

### Multivariate analysis

Different *Microtea* species were classified by group average linkage algorithm of cluster analysis constructed on a Gower similarity matrix (Gower 1971) based on thirteen characters including general morphology (life history, pubescence, leaves) and reproductive traits. This approach recognizes the species grouping based on similar characters, but does not provide a true phylogenetic context. The reliability of grouping was assessed at the level *p*<0.05 using SIMPROF algorithm (Clarke 1993, Clarke and Warwick 2001). Calculations were performed using PRIMER 6.1.6 statistical software (Clarke and Gorley 2006).

## Results

### Phylogenetic analysis of matK region

**Figure 1.**
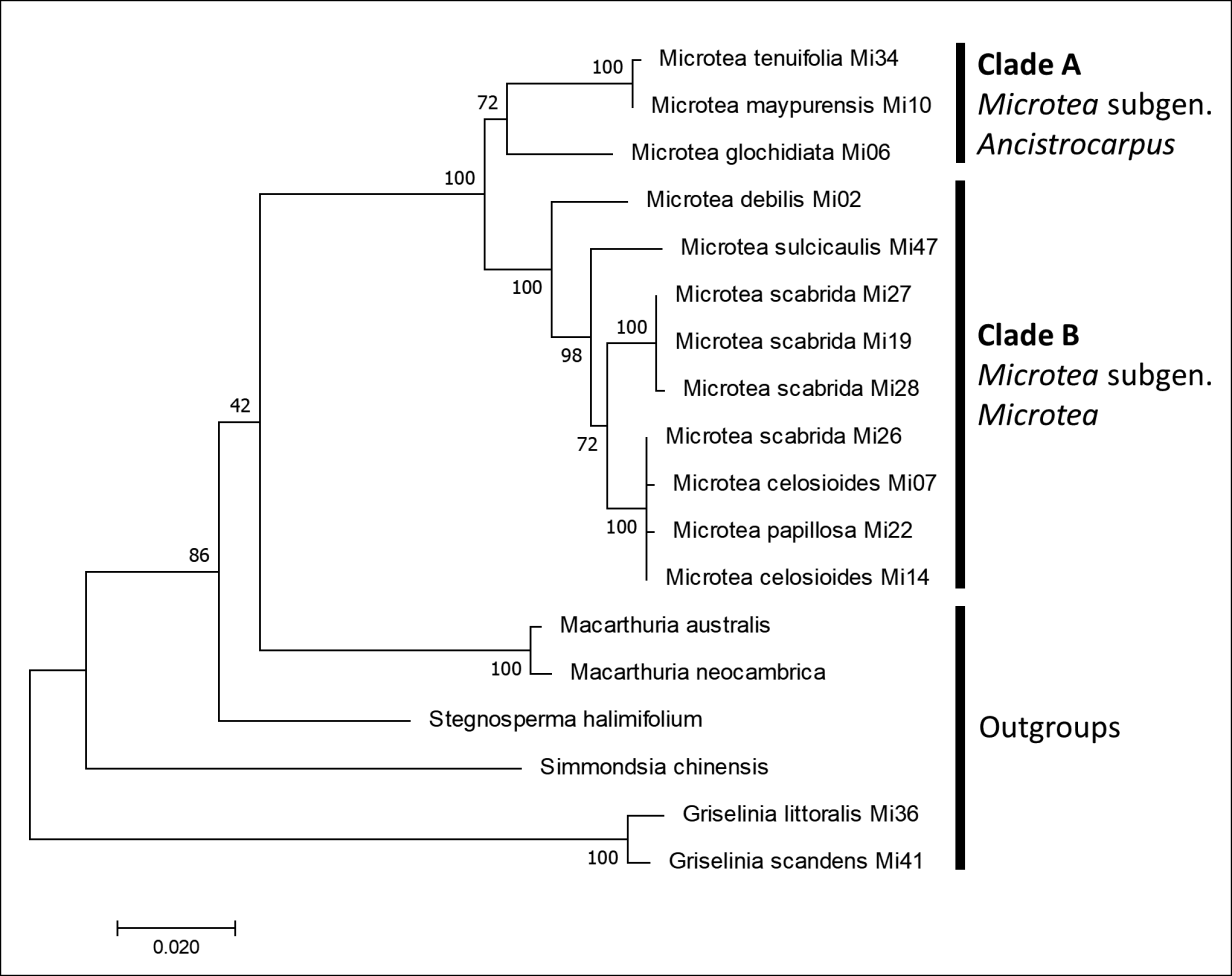
The phylogenetic tree from maximum likelihood analysis of *mat*K region sequences. The tree with the highest log likelihood (-2983.53) is shown. The analysis involved 18 nucleotide sequences. Codon positions included were 1^st^+2^nd^+3^rd^+Noncoding. There were a total of 828 positions in the final dataset.

The most representative phylogenetic analysis based on cpDNA (*mat*K) dataset shows the monophyly of the genus *Microtea*, which is divided into two well-supported clades (Fig. 1): clade A consisting of *M.glochidiata* + *M.maypurensis* / *M.tenuifolia*, and clade B comprising the remaining species included in the analysis (*M.debilis*, *M.celosioides*, *M.scabrida*, *M.sulcicaulis*, and *M.papillosa*). In clade B, *M.debilis* is a sister to the remaining species. The position of all *Microtea* species is considered to be close to *Macarthuria* (core Caryophyllales). Three clades – *Macarthuria*, *Stegnosperma* and *Microtea*, even if not fully represented in the trees based on different phylogenetic markers – occupy a basal position within the core Caryophyllales (Brockington et al. 2009, Schäferhoff et al. 2009, Sukhorukov et al. 2015). The tree based on the *mat*K region that includes these three basal lineages suggests the monophyly of *Microtea* (Fig. 1).

### Phylogenetic analysis of ITS region

**Figure 2.**
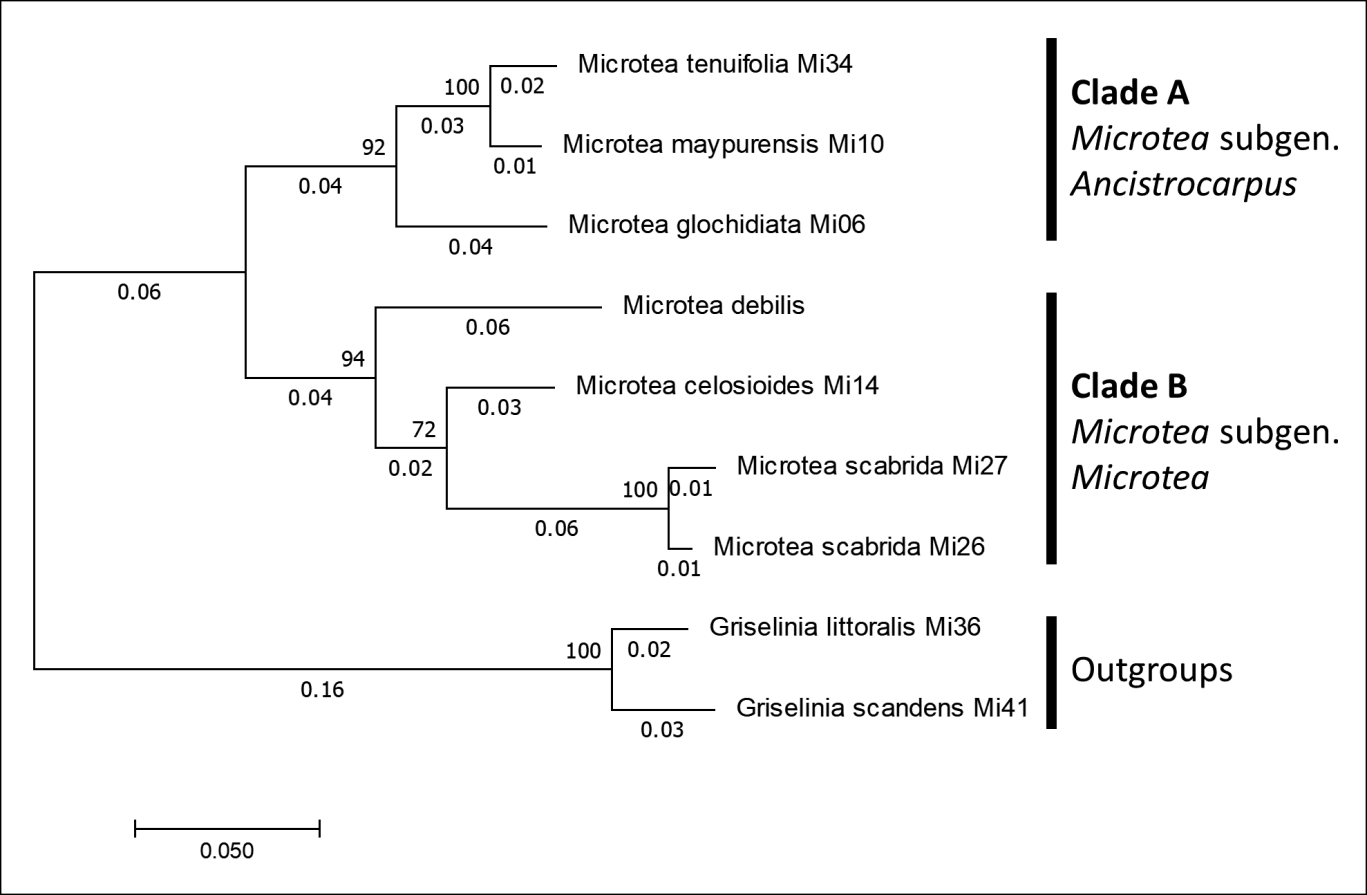
The phylogenetic tree from maximum likelihood analysis of ITS region sequences. The tree with the highest log likelihood (-2848.78) is shown. The analysis involved 9 nucleotide sequences. There were a total of 754 positions in the final dataset.

Based on the nrDNA tree (Fig. 2), the clade A (*M.glochidiata* + *M.maypurensis* / *M.tenuifolia*) is a sister to the clade B comprising the rest of the species studied (*M.debilis* + *M.celosioides* / *M.scabrida*) with good statistical support. However, only a limited number of species were included in the ITS dataset.

Based on both matK and ITS phylogenetic analyses, subgeneric status was assigned to two major clades: clade A (*M.glochidiata* + *M.maypurensis* / *M.tenuifolia*) represents M.subgen.Ancistrocarpus stat. nov., and clade B represents the type subgenus (M.subgen.Microtea).

### Carpological investigations

**Figure 3.**
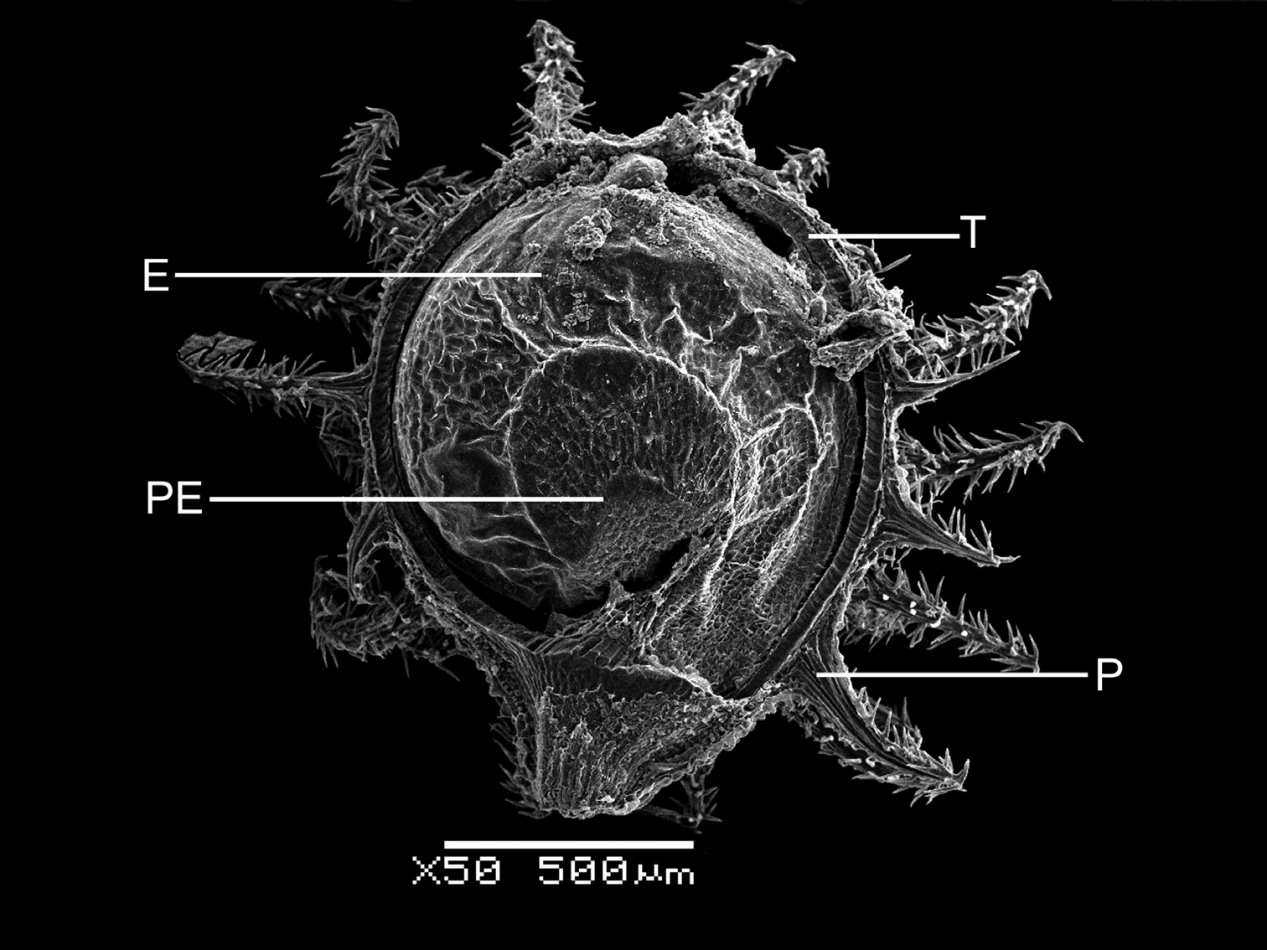
Cross-section of the fruit of *Microteaglochidiata*, showing the embryo and perisperm covered by the tegmen (Brazil, Bahia, Tucano Mun., 20 Feb 1992, *A.M. de Carvalho & D.J.N. Hind 3841*, PACA). Abbreviations: P – pericarp with plumose outgrowths, T – testa of the seed coat (tegmen covers perisperm and embryo), PE – perisperm, E – embryo. Magnification – 50×.

**Figure 4.**
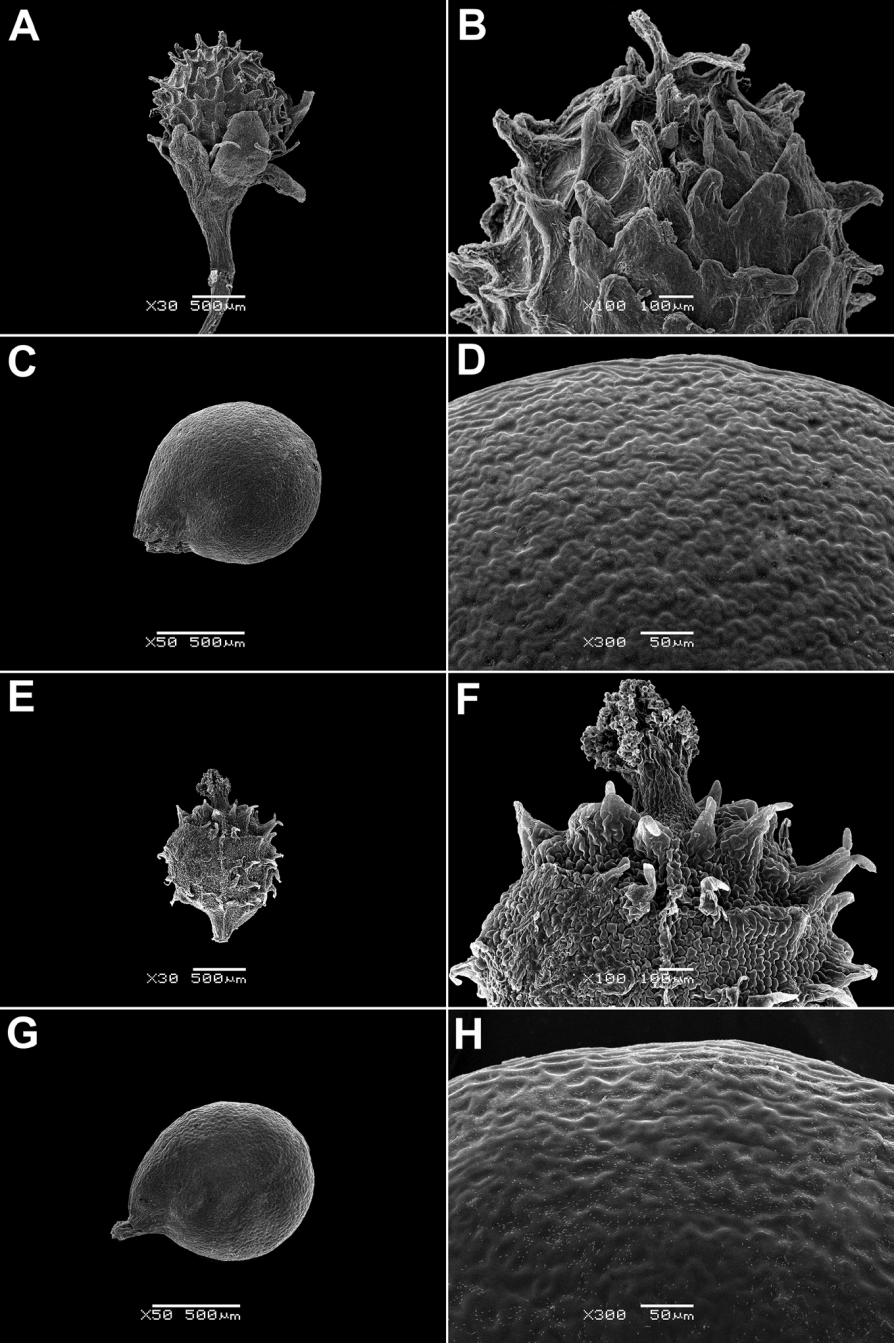
Fruits and seeds of *Microteadebilis* and *M.celosioides*: **A, B** fruit of *M.debilis*, enclosed in the perianth (St. Lucia, Soufrière, 1958, *G.R. Proctor 17789*, BM000019256) **C, D** seed of *M.debilis* (Honduras, nr Cangrejal river, foothills of Ceiba, 29 Jul 1938, *T.G. Yuncker et al. 8674*, G) **E, F** fruit of *M.celosioides* (Retiro das Pedras, Brumadinho, 14 Dec 1998, *J.R. Stehmann & C.E.S. Ferreira 2399*, PACA) **G, H** seed of *M.celosioides* (Retiro das Pedras, Brumadinho, 14 Dec 1998, *J.R. Stehmann & C.E.S. Ferreira 2399*, PACA). Magnification: **A, E** – 30×, **B, F** – 100×, **C, G** – 50×, **D, H** – 300×.

**Figure 5.**
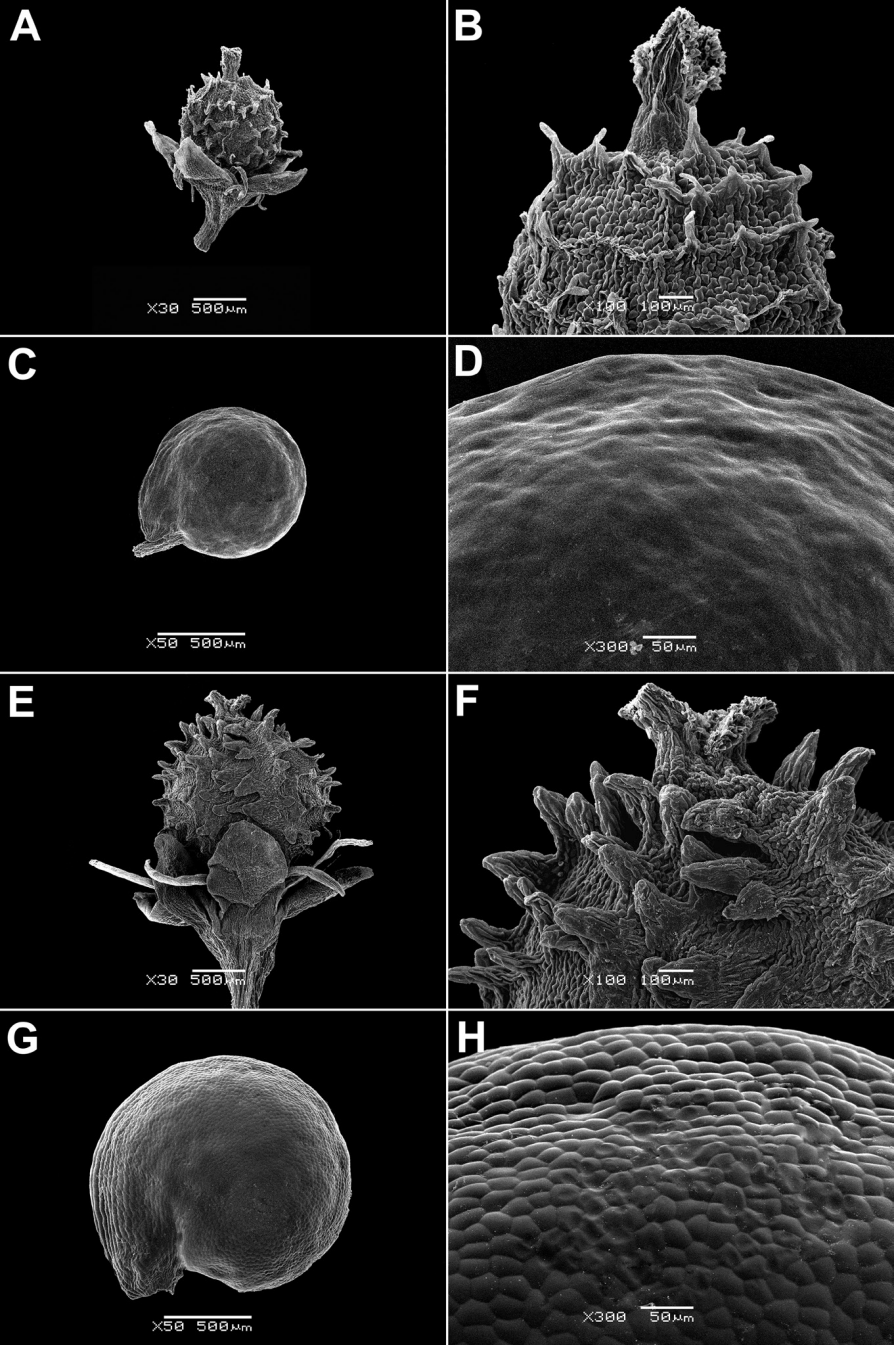
Fruits and seeds of *Microteapapillosa* and *M.scabrida*: **A, B** fruit of *M.papillosa*, enclosed in the perianth (Diamantina Mun., Estrada Conselheiro Mata, 11 Apr 1982, *L. Rossi et al. 3322*, PACA) **C, D** seed of *M.papillosa* (Diamantina Mun., Estrada Conselheiro Mata, 11 Apr 1982, *L. Rossi et al. 3322*, PACA) **E, F** fruit of *M.scabrida*, enclosed in the perianth (Paraguay, Alto Paraná, 1909, *K. Fiebrig 5468*, M) **G, H** seed of *M.scabrida* (Paraguay, Alto Paraná, 1909, *K. Fiebrig 5468*, M). Magnification: **A, E** – 30×, **B, F** – 100×, **C, G** – 50×, **D, H** – 300×.

**Figure 6.**
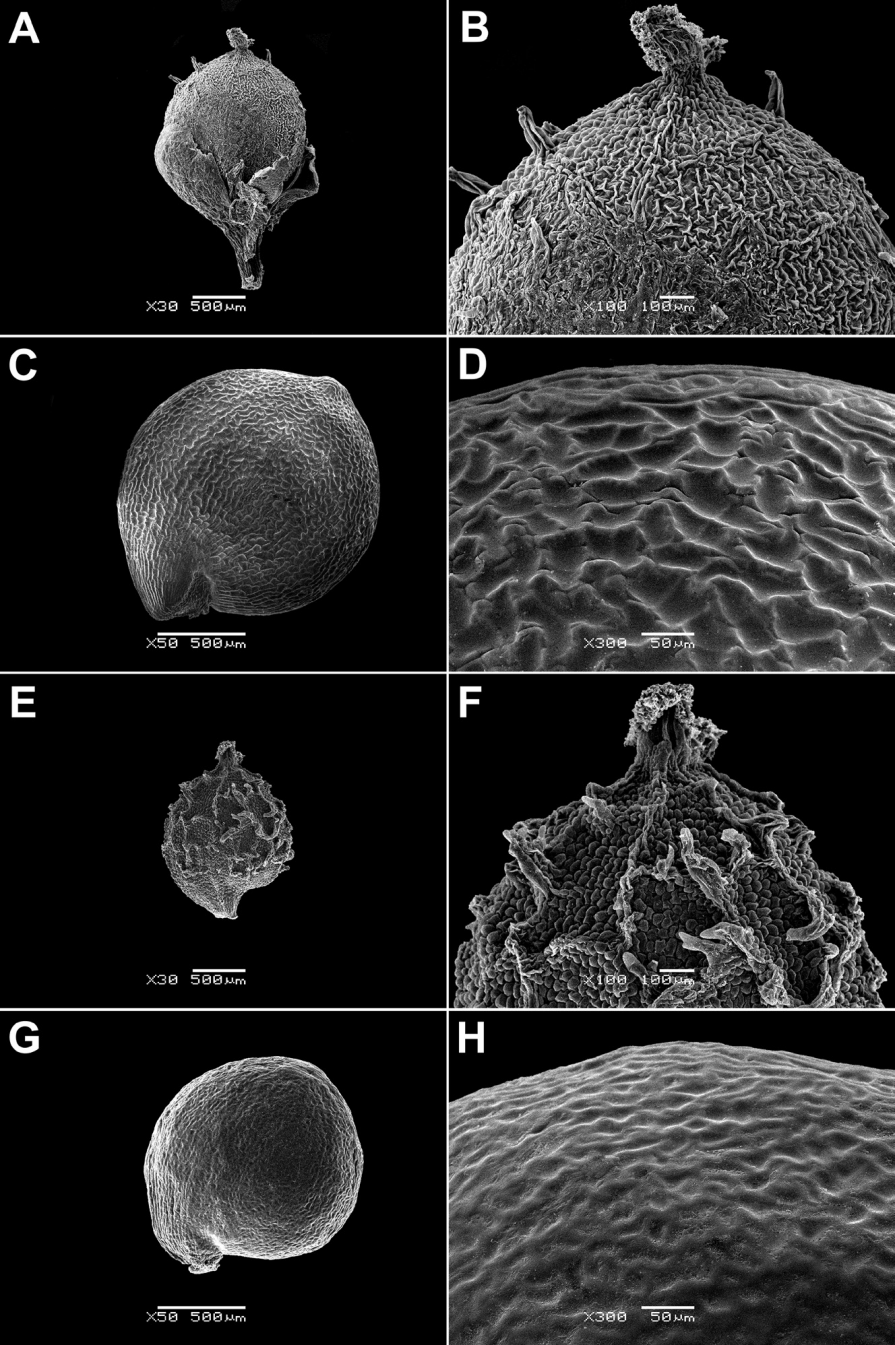
Fruits and seeds of *Microteasulcicaulis* and *M.bahiensis*: **A, B** fruit of *M.sulcicaulis*, enclosed in the perianth (Paraguay, Caazapá Dept., Tavai, 7 Dec 1988, *F. Mereles 2122*, G) **C, D** seed of *M.sulcicaulis* (Paraguay, Caazapá Dept., Tavai, 7 Dec 1988, *F. Mereles 2122*, G) **E, F** fruit of *M.bahiensis* (Brazil, Bahia state, Salvador, Dunas de Itapuã, nr Hotel Stella Maris, N from Condomínio Alamedas da Praia, 8 Jun 1993, *P. de Queiroz 3211*, PACA) **G, H** seed of *M.bahiensis* (Brazil, Bahia state, Salvador, Dunas de Itapuã, nr Hotel Stella Maris, N from Condomínio Alamedas da Praia, 8 Jun 1993, *P. de Queiroz 3211*, PACA). Magnification: **A, E** – 30×, **B, F** – 100×, **C, G** – 50×, **D, H** – 300×.

**Figure 7.**
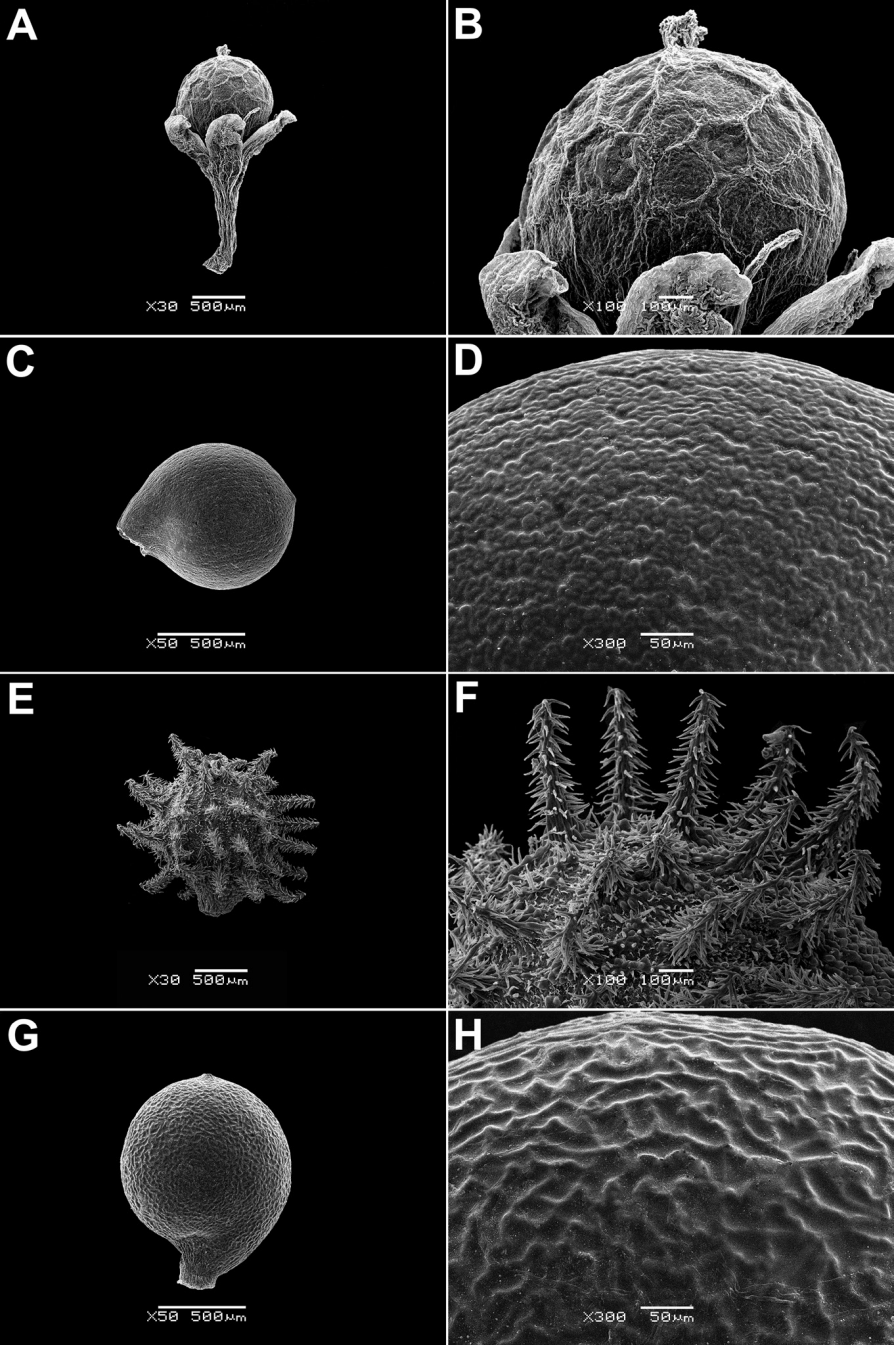
Fruits and seeds of *Microteaportoricensis* and *M.glochidiata*: **A, B** fruit of *M.portoricensis*, enclosed in the perianth (Puerto Rico, Cabo Rojo, 1864, *Grosourdy 13*, P04598159) **C, D** seed of *M.portoricensis* (Puerto Rico, Cabo Rojo, 1864, *Grosourdy 13*, P04598159) **E, F** fruit of *M.glochidiata* (Brazil, Maranhão, Barao do Grajau, 21 Jan 2012, *R.M. Harley et al. 56455*, K) **G, H** seed of *M.glochidiata* (Brazil, Maranhão, Barao do Grajau, 21 Jan 2012, *R.M. Harley et al. 56455*, K). Magnification: **A, E** – 30×, **B, F** – 100×, **C, G** – 50×, **D, H** – 300×.

**Figure 8.**
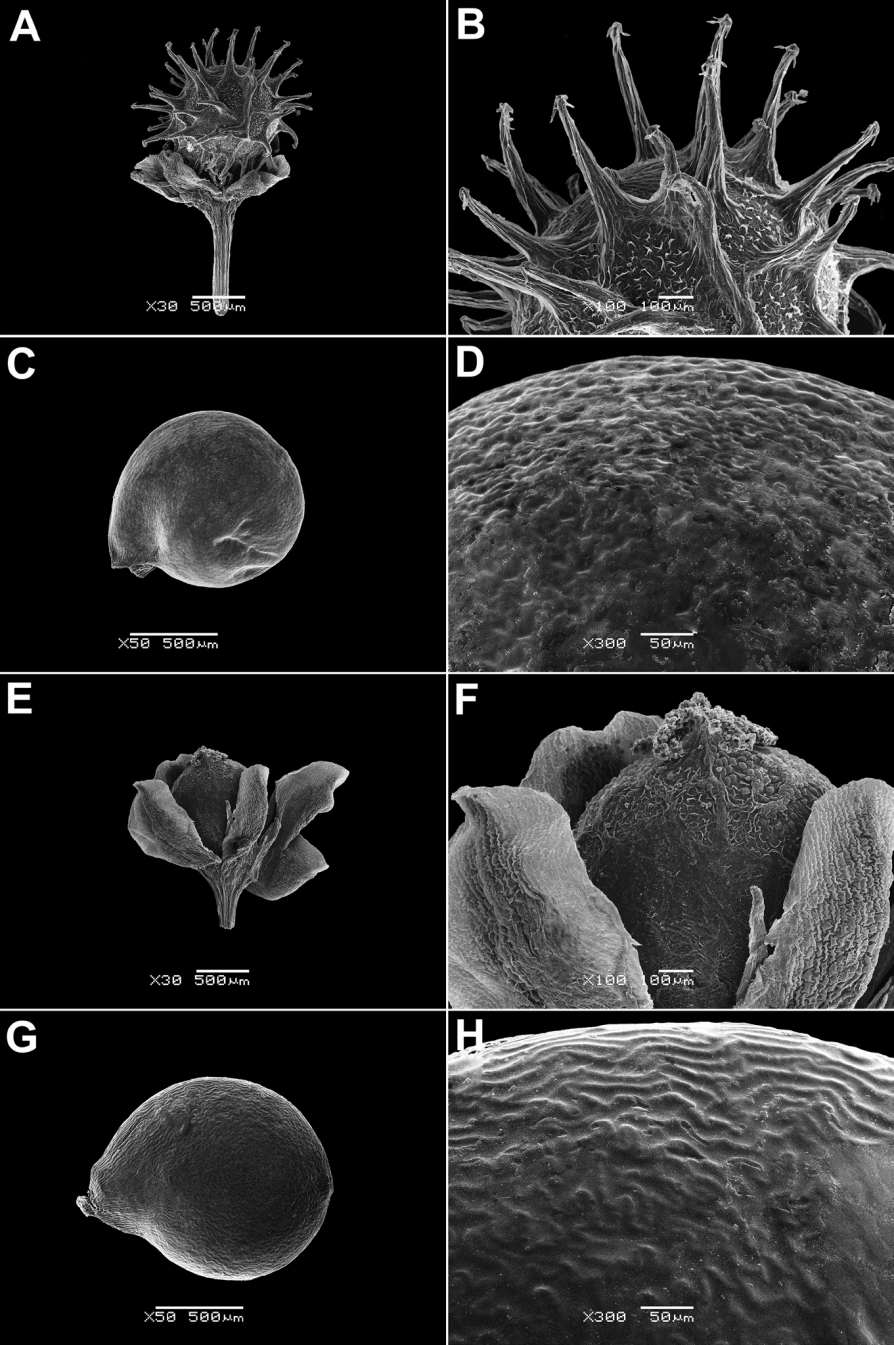
Fruits and seeds of *Microteamaypurensis* and *M.tenuifolia*: **A, B** fruit of *M.maypurensis*, enclosed in the perianth (Bolivia, La Paz Dept., Beni river, Jul 1886, *H.H. Rusby 1379*, LE) **C, D** seed of *M.maypurensis* (Bolivia, La Paz Dept., Beni river, Jul 1886, *H.H. Rusby 1379*, LE) **E, F** fruit of *M.tenuifolia* enclosed in the perianth (Brazil, Minas Gerais, Serrra das Vertentes, Jun 1893, *A. Glaziou 20437*, B) **G, H** seed of *M.tenuifolia* (Brazil, Jacobina Mountains in Bahia, 1836, *Blanchet 2588*, P00798998). Magnification: **A, E** – 30×, **B, F** – 100×, **C, G** – 50×, **D, H** – 300×.

**Figure 9.**
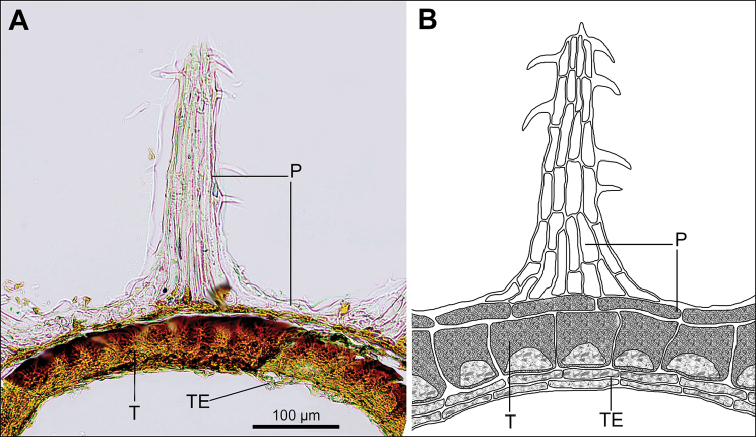
Cross-section of *Microteaglochidiata* fruit (Brazil, Bahia, Tucano Mun., 20 Feb 1992, *A.M. de Carvalho & D.J.N. Hind 3841*, PACA). **A** image of the cross-section **B** schematic representation. Abbreviations: P – pericarp; T – testa; TE – tegmen.

In all the species studied the fruit is one-seeded (Fig. 3), and the pericarp surface is reticulate (Figs 4–8, A, B, E, F). The finger-shaped (echinate) outgrowths over the entire fruit surface are present in almost all species (Figs 3; 4A, B; 5–6A, B, E, F; 7E, F; 8 A, B) except *M.portoricensis* (Fig. 7A–B), but they are usually scattered or even obscure in *M.tenuifolia* (Fig. 8E–F) and in some specimens of *M.celosioides* (Fig. 4E–F). In *M.glochidiata* they can reach 0.65 mm in length (Fig. 7E–F). The echinate outgrowths may be covered by large horizontal unicellular papillae (plumose outgrowths: *M.glochidiata*, Fig. 7E–F), or each outgrowth is terminated by a group of 2–4 recurved (hooked) hairs (*M.maypurensis*, Fig. 8A–B). Such plumose or hooked outgrowths clearly assist epizoochorous dispersal. Two thick styles are characteristic for the species forming clade B (*M.bahiensis*, *M.celosioides*, *M.debilis*, *M.papillosa*, *M.portoricensis*, *M.scabrida*, *M.sulcicaulis*), and those in the remainder of the genus (clade A: *M.glochidiata*, *M.maypurensis*, *M.tenuifolia*) possess three to five thin styles.The pericarp consists of several layers; the cells of the innermost layers are usually filled with tannins (Fig. 9 A, B). The pericarp outgrowths emerge from the mesophyll and consist of several prosenchymatous cell layers.

The seeds are spherical and black, with a rugose (Figs 4–8C, D, G, H) or slightly alveolate (*M.sulcicaulis*: Fig. 6D) surface, and are basally inserted on the fruit wall. The seed coat of all species consists of a thick (40–50 µm) exotestal layer, with outer cell walls much thicker than the arch-like protoplast (Figs 3; 9), and 1–2 barely visible layers of tegmen with bar-thickenings of its cell walls. The cells of the exotesta (Fig. 9) are dark brown due to the presence of unstructured tannin-like substances, but without additional stalactite-shaped deposits (vertical or oblique depositions of tannins originating from the outer cell walls). The annular embryo occupies a peripheral position in the seed, and is located vertically. The perisperm is abundant.

All *Microtea* species share the reticulate pericarp surface (regardless of the presence or absence of echinate outgrowths) and the rugose or slightly alveolate seed surface. The fruit and seed structure of *Microtea*, namely the homocellular pericarp consisting of several layers, seed coat with much thicker testa and barely noticeable tegmen with bar-thickenings of the cell walls, vertical embryo position in one-seeded fruits, and abundant perisperm, is typical for the core Caryophyllales (Sukhorukov et al. 2015, 2018).

### Diagnostic characters in *Microtea* and multivariate analysis

All characters discovered in *Microtea* species are summarized in Table 2.

**Table 2.** Characters of *Microtea* species.

	**Species/character**	*** M. bahiensis ***	*** M. debilis ***	*** M. celosioides ***	*** M. glochidiata ***	*** M. maypurensis ***	*** M. papillosa ***	*** M. portoricensis ***	*** M. scabrida ***	*** M. sulcicaulis ***	*** M. tenuifolia ***
1	Life history: 0 – annual or rarely biennial; 1 – upright perennials with a taproot or caudex; 2 – perennial lianas	1	0	0	0	0	1	0	2	1	0
2	Pubescence: 0 – (almost) glabrous; 1 – papillate	0	0	0	0	0	1	0	0	0	0
3	Bracteoles. 0: absent; 1: present	1	0	1	1	1	1	0	1	1	1
4	Flower arrangement. 0: solitary (inflorescence is a spike); 1: two or three in clusters (thyrsoid inflorescence)	0	0	0	1	0	0	0	0	0	0
5	Presence of pedicel. 0: flowers sessile or subsessile (pedicel up to 1.3 mm); 1: pedicel 1.35–3.0 mm	0	0	0	1	1	0	0	0	0	1
6	Perianth segments. 0: oblong or ovoid; 1: roundish	0	0	0	1	0	0	0	0	0	0
7	Number of perianth segments. 0: always five; 1: four or five (varying)	0	0	0	0	0	0	1	0	0	0
8	Number of stamens. 0: four or five; 1: more than five (usually seven or eight)	1	0	1	1	1	1	0	1	1	1
9	Number of stigmas. 0: two, rarely three; 1: three to five	0	0	0	1	1	0	0	0	0	1
10	Shape of stigmas. 0: thick; 1: filiform	0	0	0	1	1	0	0	0	0	1
11	Diameter of fruit body (without stigmas and outgrowths if the latter are present). 0: 0.9–1.1 mm; 1: 1.1–1.3 mm; 2: 1.4–2.0 mm	1	1	1	0	0	1	0	2	2	0
12	Fruit/perianth ratio. 0: fruit longer than perianth (protruding); 1: fruit equal to perianth (fruit not protruding)	0	0	0	0	0	0	0	0	0	1
13	Pericarp. 0: without any projections, reticulate; 1: with scattered, thick and simple outgrowths; 2: with abundant thick outgrowths; 3: with fimbriate (plumose) projections; 4: with projections terminating in hooks	1	2	1	3	4	1	0	1	1	0

**Figure 10.**
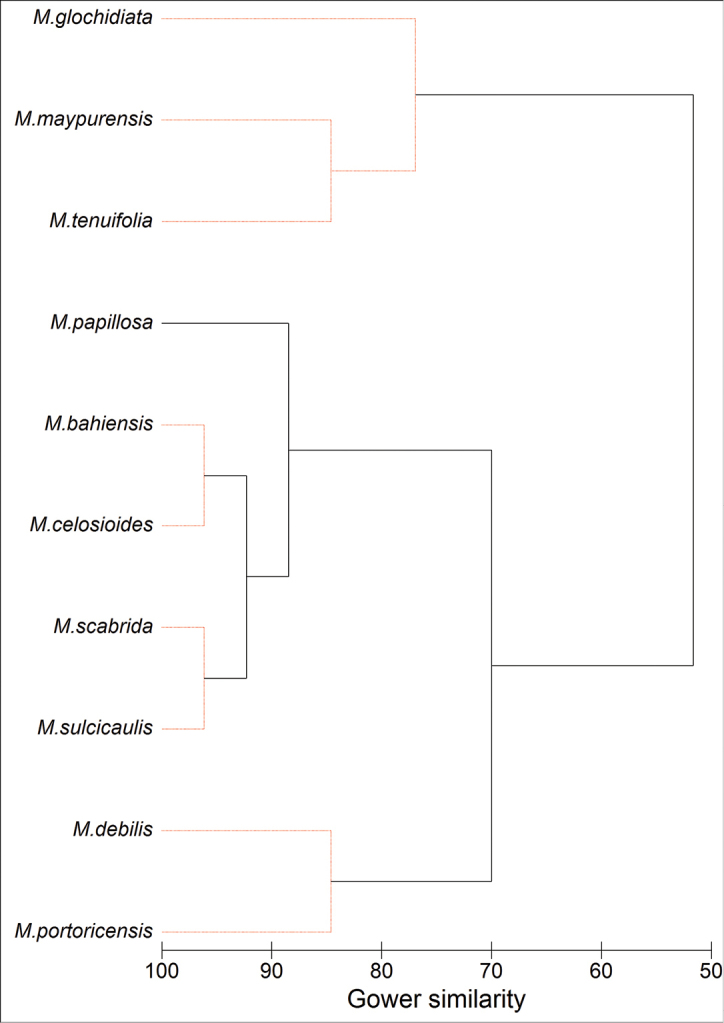
Classification of *Microtea* species by group average linkage algorithm of cluster analysis based on 13 characters. Black branches connect significantly (P < 0.05) different groups, red branches – insignificantly different groups.

The results of cluster analysis of the characters suggest the existence of five significantly different groups within the *Microtea*, these branches being highlighted in black colour (Fig. 10): (1) *M.glochidiata*–*M.maypurensis*–*M.tenuifolia*, (2) *M.debilis*–*M.portoricensis*, (3) *M.scabrida–M.sulcicaulis*, (4) *M.celosioides*–*M.bahiensis*, and (5) *M.papillosa*. The groups are significantly (p<0.05) distinguished on different levels of Gower’s index. Clusters (2–5) correspond with clade B in the phylogenetic analysis. The most prominent distinctions are observed between three major clusters consisting of (1) *M.glochidiata*, *M.maypurensis*, *M.tenuifolia*; (2) *M.debilis*–*M.portoricensis*; and (3) *M.papillosa*, *M.bahiensis*–*M.celosioides*, *M.scabrida*–*M.sulcicaulis*. Cluster (1) corresponds with clade A in the phylogenetic analyses (Figs 1, 2), and this group is clearly distant from the remaining species due to character sets 4, 5, 9 and 10. Cluster (2) comprises the species without bracteoles (character 3, state 0), and cluster 3 unites the rest of the genus with the similar sets of characters 3, 4, 5, 8, 9, 10, and 12.

### Taxonomy

#### Artificial key to the *Microtea* species

**Table:** 

1	Leaning or twining perennial herb up to 150 cm; leaf blades basally truncate	**4. *M.scabrida***
–	Smaller herbs or dwarf subshrubs up to 100 cm; leaves cuneate	**2**
2	Stems decumbent; each flower supported by a bract; bracteoles not present; stamens 4–5; annuals with obovate leaves	**3**
–	Stems usually erect; each flower supported by a bract and two transverse bracteoles; stamens 5–8; annuals or perennials with leaves not obovate	**4**
3	Pericarp with echinate (finger-shaped) outgrowths	**1. *M.debilis***
–	Pericarp not echinate (its surface reticulate)	**7. *M.portoricensis***
4(2)	Perennials or annuals with shortly but densely papillate stem and leaves	**3. *M.papillosa***
–	Perennials or annuals with glabrous stems, or with leaves papillate at margins and mid-ribs	**5**
5	Perennial herb or dwarf subshrub with well-expressed caudex and rosulate leaves; cauline leaves short (up to 2.0 cm)	**6. *M.bahiensis***
–	Annuals or perennial herbs (in latter case without a caudex); cauline leaves usually larger (2.5–12.0 cm)	**6**
6	Flowers 1–6 per node; pericarp outgrowths plumose	**8. *M.glochidiata***
–	Flowers 1(2) per node; pericarp outgrowths (if present) glabrous or hooked at apices	**7**
7	Flowers 1(2) per node; pericarp outgrowths hooked at apices	**9. *M.maypurensis***
–	Flowers always one per node; pericarp outgrowths (if present) not hooked	**8**
8	Perennial herb; leaves lanceolate or narrowly oblong, usually appressed to the stem, stiff; fruit 1.75–2.0 mm long	**5. *M.sulcicaulis***
–	Annuals, biennials; leaves filiform to oblong, not appressed to the stem, not stiff; fruit less than 1.5 mm long	**9**
9	Pedicels 1.35–1.7(2.5) mm long; fruit not protruding or slightly protruding from the perianth; pericarp smooth, verrucous or with barely visible outgrowths; leaves usually filiform or lanceolate	**10. *M.tenuifolia***
–	Pedicels up to 1 mm long; fruit twice the length of the perianth; pericarp with short finger-shaped outgrowths; leaves narrowly lanceolate to oblong (rarely ovoid)	**2. *M.celosioides***

##### Gen. Microtea

Taxon classificationPlantaeCaryophyllalesMicroteaceae

Sw., Prodr. [O.P.Swartz]: 53 (1788).

Microtea debilis Sw. (type species)
Schollera Rohr, Skr. Naturhist.-Selsk. 2: 210 (1792), nom. illegit., non Roth (1788).
Microteasubgen.Schollera (Rohr) H.Walter, Pflanzenr. (Engler) 39: 127 (1909), nom. inval. (Art. 22.2). Type species: M.debilis Sw. Note: Vahl (1792) established that a new generic name, Schollera was based on the plant that he considered conspecific with Microteadebilis. The type of Schollera Rohr is therefore that of Microteadebilis (Turland et al. 2018: Art. 10.2).
Ancistrocarpus Kunth, Nov. Gen. Sp. [quarto] 2: 186 (1817). Type species: A.maypurensis Kunth (≡Microteamaypurensis (Kunth) G.Don).
Potamophila Schrank, Pl. Rar. Hort. Monac. 2: tab. 63 (1821) nom. illegit., non R.Br. (1810). Type species: P.parviflora Schrank (=Microteamaypurensis (Kunth) G.Don).
Ceratococca Willd. ex Roem. & Schult., Syst. Veg., ed. 15, 6: LXX (1820). Type species: C.maypurensis Humb. & Bonpl. ex Roem. & Schult. (=Microteamaypurensis (Kunth) G.Don).
Aphananthe Link, Enum. Hort. Berol. Alt. 1: 383 (1821), nom. rej. Type species: A.celosioides (Spreng.) Link (≡Microteacelosioides (Spreng.) Moq. ex Sennikov & Sukhor.). Note: The generic name Aphananthe Link is rejected in favour of its later homonym, Aphananthe Planch. (Cannabaceae).

###### Description of the genus.

Annuals, perennial herbs, rarely dwarf subshrubs; stems angulate, glabrous or papillate; leaves alternate, sessile or pedunculate, entire, filiform to ovate or obovate, cuneate or truncate, apically mostly acuminate, a persistent leaf rosette usually present, cauline leaves resembling the rosulate leaves or much shorter; inflorescence a spike or thyrsoid; pedicel inconspicuous or up to 3 mm long; flowers actinomorphic, bisexual, subtended by a hyaline bract and two similar bracteoles, sometimes bracteoles absent; perianth of (4)5 glabrous segments or lobes, green, white or yellowish; stamens (4)5 in alternisepalous position, or 6–8 (in both antesepalous and alternisepalous positions), anthers 0.15–0.30 mm, introrse, thecae globose, pollen grains pantoporate; ovary roundish; style not present or very short, stigmas 2–5; fruit nut-like, single-seeded, dry; pericarp projections (if present) not evident in flowering condition, pericarp at fruiting stage reticulate, mostly having finger-shaped outgrowths (emergences) that can be plumose (with additional smaller hair-like projections) or hooked at their apices; seeds spherical, black, with rugose or alveolate surface, with annular embryo located vertically and abundant perisperm.

Ten species distributed in the (sub)tropics of the Americas; two – *M.debilis* and *M.maypurensis* – are considered as aliens in the humid tropics of Africa (Cameroon) and Asia (Indonesia), respectively.

#### Taxonomic synopsis of *Microtea*

##### Microtea subgen. Microtea

Taxon classificationPlantaeCaryophyllalesMicroteaceae

Microteasubgen.Moquinia Nowicke in Ann. Missouri Bot. Gard. 55: 349 (1968). Type: M.paniculata Moq. (=M.celosioides (Spreng.) Moq. ex Sennikov & Sukhor.).

###### Description of the subgenus.

Annuals, perennial herbs or dwarf subshrubs; bracteoles present or absent; pedicels inconspicuous or very short (up to 1.3 mm long); flowers single per node (inflorescence a spike); stigmas 2(3), thick. The species are distributed across the (sub)tropical South America, in Central America and Antilles.

##### 1. M. debilis

Taxon classificationPlantaeCaryophyllalesMicroteaceae

Sw., Prodr. [O.P.Swartz]: 53 (1788).

M.debilisSw.var.ovata Delile ex Moq. in DC., Prodr. 13(2): 17 (1849). Lectotype (Sukhorukov, designated here): Overseas territories of France. Guadeloupe, herb. Desfontaines (P00798994!).
M.debilisvar.rhombifolia Moq. in DC., Prodr. 13(2): 17 (1849). Holotype: [without locality data] “Herb. Poiret in Herb. Moquin-Tandon” (P00798997!). Note: One specimen at K contains both varieties (M.debilisvar.ovata andM.debilisvar.rhombifolia) mounted on one sheet and identified as such by Moquin-Tandon. It contains several plant fragments with different labels. The varieties can barely be distinguished from one another.

###### Lectotype.

(designated by Howard and Howard 1982: 76): OVERSEAS TERRITORIES OF THE NETHERLANDS. St. Eustatius, *F. Masson s.n.* in Herb. Banks (BM000019252!). Note. The species was described from the West Indies, and Saint Christopher Island (also known as Saint Kitts Island, St. Kitts & Nevis) was reported as the only locality in the protologue. Although the personal herbarium of Swartz is incorporated in S, he noted for some species in his book (Swartz 1788) that in such cases he used the herbarium collections in Banks’s possession (Stearn 1961, Howard and Howard 1982). The collections used by Swartz (1788) are listed in Swartz’s subsequent publication, *Flora Indiae Occidentalis*, in which the original locality of *M.debilis* was stated more accurately as the island of “St. Eustathii”, and “Masson” was indicated as the collector (Swartz 1797: 543). This specimen is the only element associated with the taxon by the original author, and consequently it was designated as the lectotype of the name (Howard and Howard 1982: 76). Although this was not indicated in the protologue, probably a further collection was available to Swartz by that time: a specimen of *M.debilis* collected by H. de Ponthieu (reportedly in Grenada) was acquired by Swartz from Banks and subsequently given to L.J. Montin, a Swedish collector of herbarium material, whose private herbarium became part of S along with that of Swartz himself (Lindman 1916).

###### Description.

**Figure 11.**
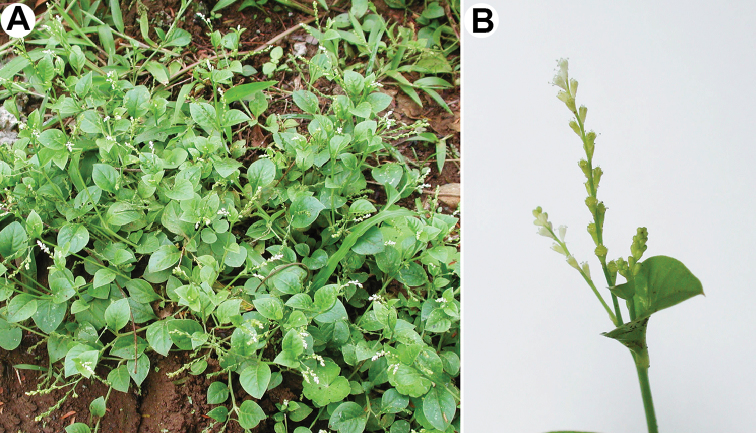
*Microteadebilis*: **A** general view of the plants (La Selva Biological Station, Costa Rica, 2001) **B** close-up of the inflorescence (La Selva Biological Station, Costa Rica, 2001). Photographs by Orlando Vargas Ramírez. See also https://sura.ots.ac.cr/florula4/find_sp3.php?key_species_code=LS001515.

Annuals, glabrous; stems decumbent, up to 30 cm (Fig. 11A); rosulate leaves up to 9 (10–12) cm, long-petiolate, obovate or oblong, mostly persistent; cauline leaves rhombic or ovate, cuneate; inflorescence a spike (Fig. 11B); flowers sessile or very shortly pedicellate (pedicels at fruiting ~1 mm); bracteoles absent; perianth segments 5, greenish, lanceolate or oblong; stamens (4)5; stigmas 2, thick; fruit roundish, 1.1–1.25 mm long and 1.0–1.2 mm wide (Fig. 4A, B), with finger-shaped outgrowths (up to 0.4 mm long); seed ~1.0 mm, with rough surface (Fig. 4D).

###### Habitat.

Sands, forest margins, or as a weed; altitudes up to 1000(1200) m a.s.l.

###### Distribution.

**Figure 12.**
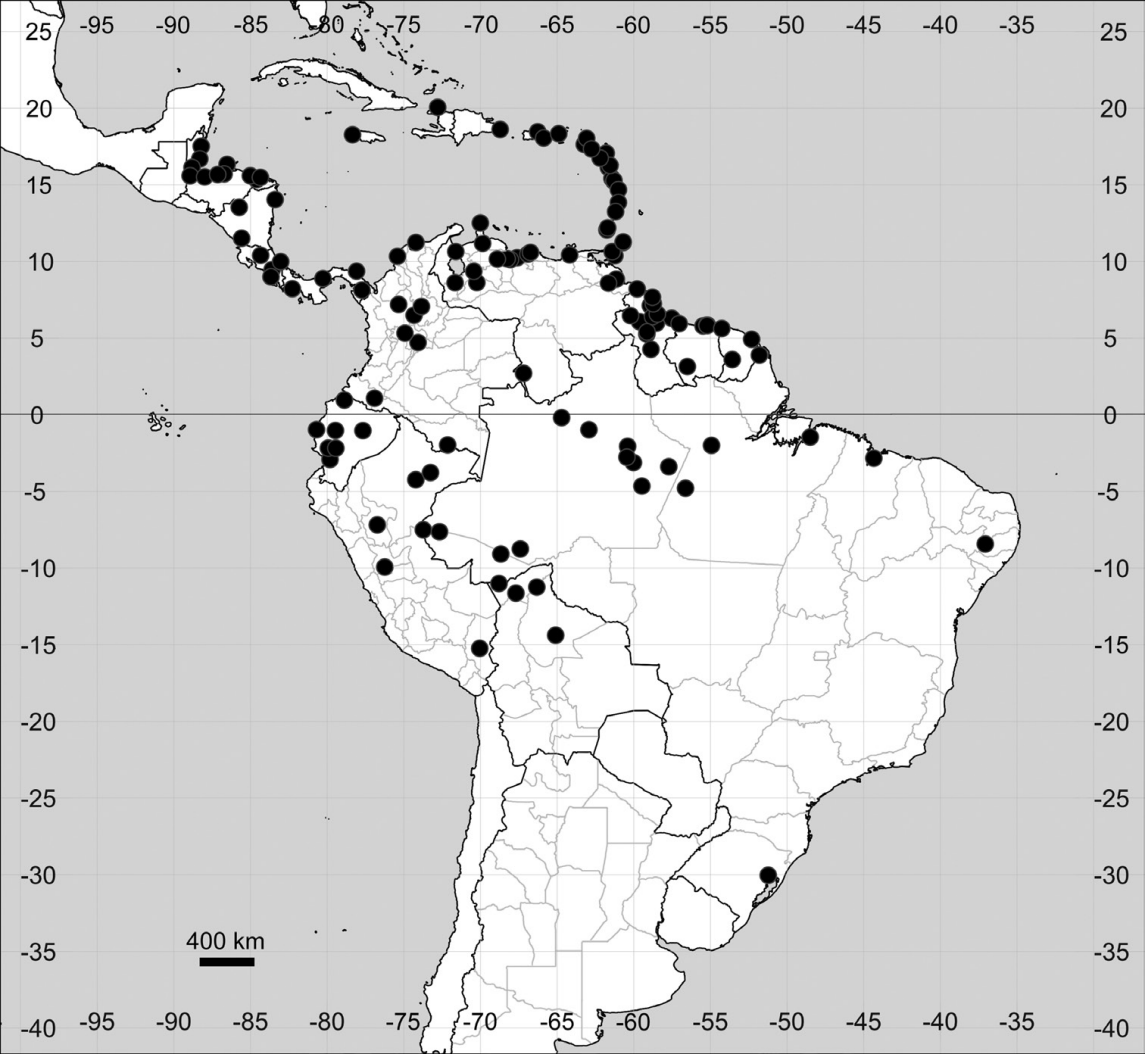
Distribution map of *Microteadebilis* in its native range.

Native to American tropics (Fig. 12).

###### Specimens examined.

**Figure 13.**
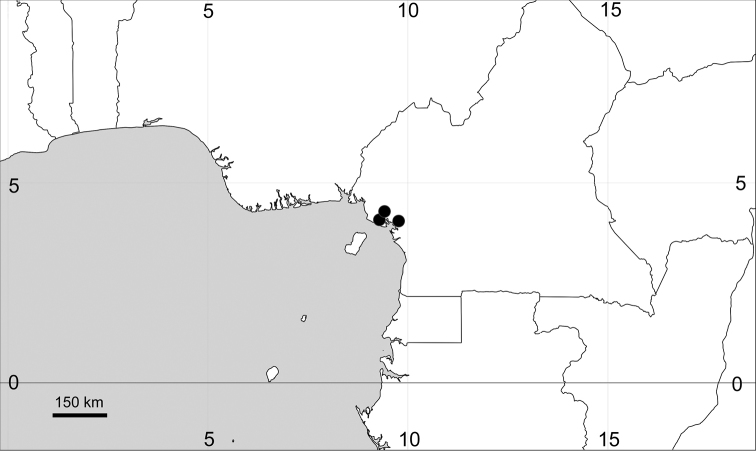
Records of *Microteadebilis* as an alien plant in Africa.

**antigua & barbuda**: Dimsdale, 29 Aug 1937, *H.E. Box 1005* (BM000019212); **belize**: Manatee Lagoon, 1905, *M.E. Peck 42* (K); 22 mile of Stann Creek river, 250 ft, 3 July 1932, *W.A. Schipp 286* (K); Bright Lookout Bank, Sibun river, 12 Jan 1935, *P.H. Gentle 1487* (K); Toledo distr., 28 Oct 2005, *C. Whitefoord & V. Quiroz 106292* (BM000895516); **bolivia**: La Paz, Upper Rio Beni, Dec 1906, *J.W. Evans 54* (BM); Rio Acre, Jan 1912, *E. Ule 9363* (G, K); Beni Dept., provinces Ballivián & Yacuma, 27 May 1985, *I. Guareco 477* (M); Beni Dept., provinces Ballivian & Yacuma, comunidad Charaton, 11 Mar 1995, *E. Rivero 293* (M); **brazil**: **Acre**: Sena Madureira Mun., 10 Oct 1968, *G.T. Prance & al. 7958* (P05197144, U1473418); Serra do Moa, 24 Apr 1971, *G.T. Prance et al. 12403* (K); Sena Madureira to Rio Branco, 10 Oct 1968, *G.T. Prance et al. 7958* (K); Cruzeiro do Sul, 18 Nov 2001, *T.B. Croat 85419* (NY01187675 – image!); **Amazonas**: Barra do Rio Negro [Manaus], 1850, *R. Spruce 1078* (BM000019274, M); Mun. Boca do Acre, Rio Acre, Jan 1912, *E. Ule 9363* (L1673158); Barcelos, 9 Sep 1962, *A.P. Duarte 7284* (RB00272328 – image!); Manaus, 20 Oct 1971, *P.J.M. Maas & H. Maas 498*(U1473424); Manaus Mun., Rio Cuieiras, 50 km upstream, farm of Sr. Nemerio, 2 Apr 1974, *J.C. Ongley & J.F. Ramos P21790* (K, U1473416); Rio Mapari, ca 30 km E of Borba, 24 Jun 1983, *C. Todzia et al. 2246* (K); Maués Mun., ca. 20 km E of Maués, 23 Jul 1983, *S.R. Hill 13146* (K); **Maranhão**: Rio Perizes, 12 Dec 1976, *B.G.S. Ribeiro & G.S. Pinheiro 1752* (IAN154119 – image!); **Parà**: 1908, *C.F. Baker 29* (K, M); Rio Tapajos, 29 Feb 1920, *anonym 14684* (RB00272338 – image!); Belém, 10 Nov 1942, *W.A. Archer 7794* (K); **Pernambuco**: Aliança, Rio Branco, Apr 1913, *J.G. Kuhlmann 3139* (RB00272342 – image!); **Rio Grande do Sul**: Porto Alegre, Jan 1898, *Reinedly s.n.* (E). Note: The isolated record from Rio Grande do Sul may be locally alien, since the specimen was collected near the port. **colombia: Cundinamarca Dept.**: Bogotá, 1851–1857, *J. Triana 5260* (P04598044); **Guainía Dept**.: Rio Negro, San Felipe, 28 Sep1921, *Ph.v. Luetzelburg 22249* (M); **Magdalena Dept**.: Santa Marta, 1898–1901, *H.H. Smith 1246* (BM000019264, G, E, K, L1673160, LE, P04598033); **Antioquia Dept**.: Vuelta de Acuña, 14 Jan 1918, *F.W. Pennell 3815* (K); **Bolivar Dept**.: nr Turbaco, 9 Nov 1926, *E.P. Killip & A.C. Smith 14359* (LE); **Amazonas Dept**.: Rio Putumayo, Sep/Oct 1930, *G. Klug 1647* (BM000019246, K); Rio Igara Paraná, Puerto Buenaventura, 12 Oct 1973, *C. Sastre 2484* (P04448856); **Santander Dept**.: Puerto Berrio, 6 Jun 1935, *O. Haught 1752* (BR, BM000019237); nr Barrancabermeja, 5 Mar 1967, *J. de Bruijin 1593* (WAG1166513); **Tolima Dept**.: San Sebastián de Mariquita, 25 Sep 2001, *G.C. Bernal et al. 1096* (COL000007803 – image!); **Costa Rica**: Talamanca, Mar 1894, *A. Tonduz 8712* (BM000019277, P03321182); Sipurio, Apr 1894, *H. Pittier & Th. Durand 8712* (M, P04598123); Alajuela prov., Los Chiles, 4 Aug 1949, *R.W. Holm & H.H. Iltis 933* (G, P04598124); Limón prov., Cordillera de Talamanca, 9°40'25"N, 83°01'35"W, 24 Feb 1989, *G. Herrera 2439* (BM000019239); **DOMINICA**: 1838, *Murray 344* (K); Oct 1881, *H. Eggers 563* (BR, G, L1673154, LE, LY, M, P04597992); Dubuc, 21 Jul 1983, *Assi & Portecop 16503* (P06806988); St. Patrick, 8 Feb 1986, *C. Whitefoord 5386* (BM000019281); **DOMINICAN REPUBLIC**: Higüey, Nov 1946, *R.A. Howard & E.S. Howard 9731* (P04598162); **ECUADOR**: **Esmeraldas prov**.: Timbre, 2 Jun 1955, *E. Asplund 16558* (B, G, K, P04598007); **Guayas prov**.: Balao, [without date] *Hamilton 373* (K, BM000019242); Balao, Dec 1891, *Eggers 14122* (B); Guayaquil, 1846–1849, *W. Jameson 373* (BM); nr Naranjito, Jun 1945, *W.H. Camp 3575* (P04598008); **Los Ríos prov**.: Clementina on Rio Pita, 12 Mar 1939, *E. Asplund 5253* (BR, G, K, P04598009); **Manabi prov**.: El Recreo, 1897, *H. Eggers 14927* (LE); Naranjapata, 21 Nov 1933, *H.J.F. Schimpff 505* (G, M); **Napo prov**.: Puerto Misahualli, May 1983, *W. Palacios et al. 366* (K); **FRENCH GUIANA**: St Georges, 14 May 1983, *M.F. Prévost 1356* (P04598030); Eau Claire, 3°37'N, 53°12'W, 15 Aug 1993, *S. Mori et al. 23295* (P05197089); Elahé, Abati Ti Wan, 6 Mar 1999, *M. Pignal 927* (P00176753); Cayenne, 15 May 1999, *M.-F. Prévost 3657* (P5197094, U1473414); **GRENADA** [Main Island]: 1844, *J. Goudot s.n.* (P04598036); Victoria [city], 20 Nov 1957, *G.R. Proctor 17140* (BM000019255); St. George’s, 11 May 1905, *Broadway 4366* (BR); **GUADELOUPE** (selected specimens): see type of M.debilisvar.ovata; [without exact location and date] *Bertero*, herb. *J. Gay 1820* (K); [without exact location and date] coll. *Bertero s.n.* [herb. De Candolle] (G00676754); [without exact location] 1907, *P. Duss 41* (LY); Rivage de Capesterre, 1 Apr 1943, *Questel 5064* (P04597987); **GUATEMALA**: Izabal, Aug 1870, *G. Bernoulli 877* (G, K); **GUYANA** (selected specimens): [without location] 1868, *Schomburgk 229* (BR, L1673157, P04598083); Mazaruni river, Sep 1880, *G.S. Jenman 748* (K); Mazaruni river, Aug 1889, *Jenman 5277* (BM000019244, K); Rockstone, Sep 1905, *A.W. Bartlett 8559* (K); Aruka, NW district, Nov 1915, *C.K. Bancroft & N. Persaud s.n.* (K); Tumatumari village, Jun 1921, *H.A. Gleason 18* (K); Amakura river, Northwest district, 8°10'N, 60°W, Mar 1923, *J.S. de la Cruz 3548* (K); Pomeroon district, Moruka river, Jul 1927, *J.S. de la Cruz 4601* (K); Cuyuni river, near Lower Camaria landing, 23 Nov 1929, *N.Y. Sadwith 666* (K); Mabaruma compound, 11 Jul 1934, *W.A. Archer 2256* (K); Waranama ranch, intermediate Savannahs, Berbice river, 9 Jun 1958, *Harrison 1073* (K); Potaro river, near Amatuk, 2000 ft, 10 Sep 1959, *B.A. Whitton 344* (K); [West Demerara Region] Essequibo, Henrietta, 4 Feb 1960, *J.A. Harris 143* (MHA); Royal Island, 5 May 1985, *C. Feuillet 2181* (B, P04598031); Cuyuni-Mazaruni Region, near Eping river, 5°58'N, 60°13'W, 396 m, 10 Feb 1991, *T. McDowell 3985* (K, U1473392); Pomeroon-Supenaam Region, Kabakaburi river, 8 Sep 1992, *B. Hofmann & L*. *Roberts 2443* (U1473417); Rupununi distr., Manari, 2 Aug 1995, *M.J. Jansen-Jacobs et al. 4733* (U1473413); North-West distr., Moruka river, 9 Oct 1997, *T. van Andel 1959* (U1473415); **HAITI**: Ile La Tortue [Tortuga Island], La Vallée, 21 May 1925, *E.L. Ekman 4063* (G, K); **HONDURAS**: Roatan Island, Aug 1886, *G.F. Gaumer 27* (K); Santa Barbara Dept., San Pedro Sula, May 1888, *C. Thieme 5427* (K); nr Cangrejal river, foothills of Ceiba, 29 Jul 1938, *T.G. Yuncker et al. 8674* (BM00019283, G, K); Gracias a Dios dept., Cocobila, 10 Feb 1981, *G.R. Proctor 38968* (BM000019258); Gracias a Dios dept., Rio Platano Biosphere Reserve, 30 May 1985, *S.G. Knees 2821* (BM000019248); Atlántida, La Ceiba, 26 Sep 1991, *M. Chorley 439* (BM); Gracias a Dios dept., Tawahka Asangni Biosphere Reserve, 1994, *P. House 99* (BM000833935); **JAMAICA**: Negril, 9 Mar 1908, *W. Harris 10214* (BM000019231, K, P04598156); **MARTINIQUE**: [year] 1833, *Sieber 93* (BM000019251, BR, G, M, P04597991); [without exact location] 1839, *Rivoire s.n.* (P04597989); [without exact location] 1868–1869, *Hahn 1026* (B); nr St Pierre, Jun 1879, *Cosson 811* (K); June 1913, *M. Mouret 128* (P04597990); Tivoli, 12 Dec 1944, *H. Stehle 5552* (U1473388); **MONTSERRAT**: Plymouth, 5 Feb 1959, *G.R. Proctor 19021* (BM000019257); **NETHERLANDS ANTILLES: St. Eustatius Island**, [without exact location] 1908, *I. Boldingh 89* (U1473381); **Saba Island**, Bottom city, 29 Aug 1947, *F. Arnoldo 892* (U1473385); **St. Martin**: Filipsburg to Belvedere, 18 Aug 1908, *I. Boldingh 2588* (U1473387); **NICARAGUA**: Omotepe Island, Oct 1869, *P. Lévy 241* (G, P04598125); Puerto Cabezas, 5 Oct 1978, *W.D. Stevens 10563* (MO-20480120 – image!); Wiwili Mun., 20 Jan 2006, *I. Coronado et al. 30888* (MEXU); **PANAMÁ**: Isthmus of Panama, Feb 1850, *A. Fendler 109* (K); [no exact location] Jul 1861, *J. Hayn 196* (BM000019238, K); Isla Brava, 8 Jun 1909, *E. André 350* (K); El Real, 16 Jun 1959, *W.L. Stern et al. 621* (LE); Fort San Lorenzo, 17 Dec 1966, *D. Burch et al. 1025* (K); Ailigandi, 8 Oct 1978, *B. Hammel 5041* (MEXU); **PERU**: **Huánuco Region**: Prov. Pachitea, Dept. Huanuco, Bosque National de Iparia, 6 Dec 1966, *J. Schunke 1322* (G); Panguana, Nov/Dec 2008, *G. Gerlach 136* (M); **Puno Region**: [San] Gaban, Aug 1854, *R.F. Hohenacker 2443* (G, M); **Loreto Region**: Maucallacta, Rio Paranapura, Jan 1935, *G. Klug 3959* (BM000019247, K); Loreto prov., 30 Oct 1940, *E. Asplund 14151* (G, K); Loreto dept., Rio Nanya, 7 Aug 1972, *T.B. Croat 18874* (E); Dept. Iquitos, Maynas, 19 May 1986, *M. Rimachi 8194* (BR); **San Martín Region**: Prov. Mariscal Cáceres, Dept. Tocache Nuevo, 6 Nov 1969, *J. Schunke 3585* (G); **PUERTO RICO**: nr Dorado, Mar 1922, *N.L*. *Britton et al. 6650* (NY00992870 – image!); Yabacoa, 11 Oct 1968, *R.J. Wagner 1687* (U1473389); Mun. de Patillas, 15 May 1988, *C.M. Taylor & J. Druitt 8097* (NY00992874 – image!); **ST. KITTS & NEVIS**: St. Kitts, nr Canada Estate, Sep/Oct 1901, *N.L. Britton & J.F. Cowell 275* (NY01509872 – image!); **ST. LUCIA**: St Lucia, Jun 1879, *H.B. Murray s.n.* (K); Soufrière, 1958, *G.R. Proctor 17789* (BM000019256); Anse Mamin, 21 Nov 1938, *H.E. Box 1999* (BM); **ST. VINCENT & GRENADINES**: St. Vincent [Island], 1822, *L. Guilding s.n.* (K); St. Vincent Island, 1826, *Lambert s.n.* (BR); [St. Vincent Island] pastures at Petit Bordell estate, 14 Oct 1949, *I. Velez 3341* (K); St. Vincent Island, [without date and collector] *178* (BM000019219); **SURINAME** (selected specimens): 1843, *Hostmann s.n.* (K); Paramaribo, 1851, *Wullschlaegel 445* (BR); Groningen, 10 May 1916, *J.A. Samuels 115* (K, L1673164, P04598019); Corantijnpolder nr Nieuw Nickerie, 27 Aug 1933, *J. Lanjouw 634* (K); Landsboerderij, 11 Feb 1955, *J.C. Lindeman 521* (U1473398); Zuid river, 3°20'N, 56°49'W, 30 Sep 1963, *H.S. Irwin et al. 57701* (M, P04598018); distr. Suriname, Kalpoeweg, 13 Apr 1981, *Ch. Kalpoe 16588* (U1473404); Marowijne, Bigiston, 13 Apr 2006, *T.R. van Andel & L. McIntosh 5180* (L0842725); **TRINIDAD & TOBAGO** (selected specimens): Trinidad [Island], 1826, *Sieber 134* (L1673148, LE, M, P04598179); Tobago [Island], Nov 1889, *H. Eggers 5826* (P04597985); Tobago, Roxborough, 16 Oct 1912, *W.E. Broadway 4642* (G, K); Tobago, 15 May 1913, *anonym s.n.* (P04597986); Trinidad, Imperial College of Tropical Agriculture [St Augustine], New Farm, 80 ft, 20 Feb 1958, *J.W. Purseglove 6112* (K); Trinidad, Curepe, 8 miles E of Port-of-Spain, 25 May 1975, *A. Raynal 15549* (K); Tobago, Castara, 18 Jul 1910, *W.R. Broadway 4068* (E); **VENEZUELA** (selected specimens): **Amazonas**: Dept. Casiquiare, Maroa, 25 Aug 1978, *O. Huber 2571* (K); Alto Orinoco Mun., 1 May 2005, *A. Fernández et al. 21234* (NYBG03142590 – image!); **Aragua State**: Maracay, [without date] *P.C. Vogl 20* (BR, M); **Barinas State**: Barinas to San Cristobal, 13 Mar 1964, *F.J. Breteler 3692* (WAG1166512); **Capital District**: Caracas, 1864, *Grosourdy 19* (P04598045); Cordillera de la Costa, 19 Aug 2000, *W. Meier et al. 7418* (M); Cordillera de la Costa, 1 May 2002, *W. Meier et al. 8153* (G); **Carabobo State**: Companero, 1843, *J. Linden 1338* (LE, P04598050); San Esteban, 1893–1894, *Mocquerys s.n.* (H1332163); Chirgua, 700 m, 1 Jan 1939, *A.H.G. Alston 5960* (BM000019204, LE, U1473454); Distr. Bejuma, 16 Apr 2000, *W. Meier & N. Flauger 6878* (G); **Delta Amacuro State**: Delta of Orinoco, San Antonio, 16 Feb 1911, *F.E. Bond et al. 140* (K); **Falcón State**: Rio Tucuyo, 26 Jan 1966, *J.A. Steyermark & A. Braun 94504* (M); **Mérida State**: El Vigia, [without date] *Mocquerys 981* (P05197141); **Monagas State**: Lower Orinoco, Sacupana, Apr 1896, *H.H. Rusby & R.W. Squires 77* (E, G, K, M); **Sucre State**: Cumaná, 1893–1894, *Mocquerys 814* (K, P04598035); **Trujillo State**: Trujillo city, 22 May 1971, *C. Emilin & B. de Rojas 964* (U1473456); **Vargas State**: road from La Suagra to Macuto, Apr 1854, *anonym s.n.* (K); **Yaracuy State**: Chivacoa, 6 Mar 2004, *W. Meier & S. Nehlin 10101* (G); San Felipe distr., 23 Mar 2004, *W. Meier & J.L. Escalona 10219* (B 10 0455316); **Zulia State**: Maracaibo, 1826, *Plée s.n.* (P04598048); [without exact location] 1893–1894, *Mocquerys 931* (P04598034); **VIRGIN ISLANDS** (US): St Croix, Jolly Hill, 20 Jan 1906, *C. Raunkier s.n.* (BR, P04598163); St. Thomas, Charlotte Amalie, 9 Feb 1913, *N.L. Britton et al. 470* (NYBG01509850 – image!); Note: Data were not available on the presence of the species in Cuba, in agreement with the recent treatment of *Microtea* in this country (Greuter 2002). As alien found in tropical Africa (Bamps 1974; see also Fig. 13): **CAMEROON**: Douala, sea shore, Oct 1938, *H. Jacques-Félix 2203* (P04621264); Douala, 50 m, 21 Mar 1967, *A. Meurillon 661* (K); Douala, 8 Oct 1969, *anonym 1721* (P04621263, WAG1166511); West province, Moliwe, 3 miles N of Victoria, 400 ft, 16 Aug 1969, *H. Chuml 301* (K); South-West Province, NE of Muyuka, at foot of Cameroon Mt., 26 Aug 1983, *D. Thomas 2538* (K, P05156267, P05156282, WAG1166510).

##### 2. M. celosioides

Taxon classificationPlantaeCaryophyllalesMicroteaceae

(Spreng.) Moq. ex Sennikov & Sukhor., comb. nov.

urn:lsid:ipni.org:names:77193071-1

**Figure 14.**
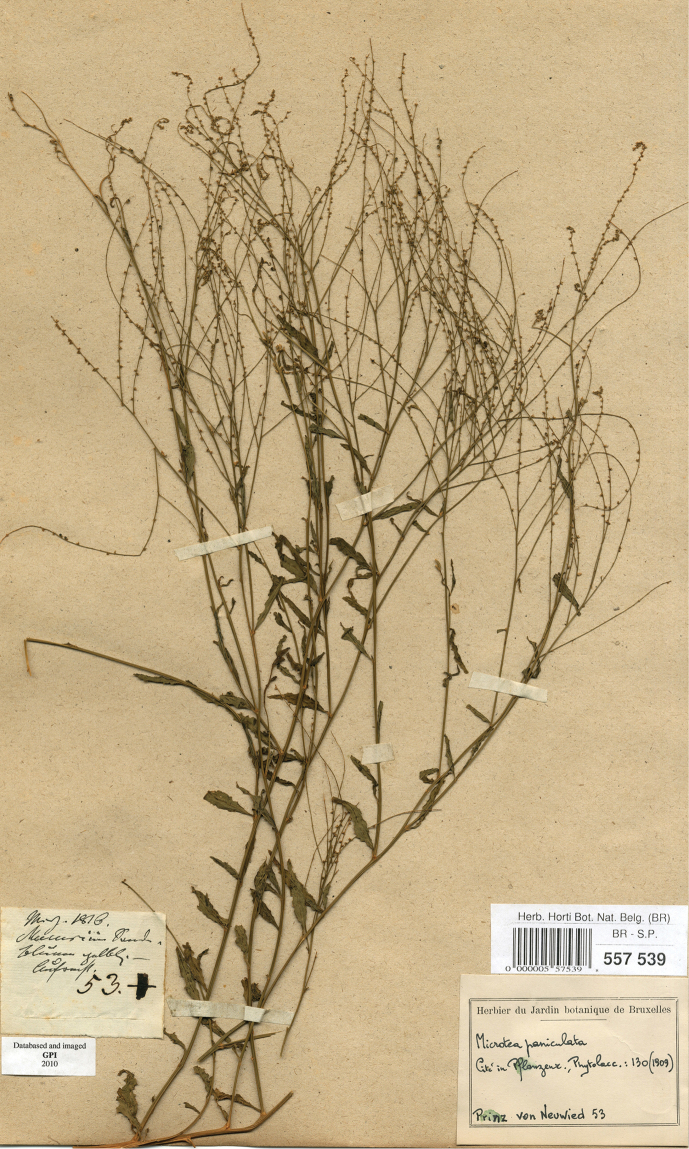
Neotype of *Galeniacelosioides* (BR0000005575398).

Galeniacelosioides Spreng., Nov. Prov.: 18 (1818). ≡Aphananthecelosioides (Spreng.) Link, Enum. Hort. Berol. Alt. 1: 383 (1821). Neotype (designated here by Sennikov & Sukhorukov): BRAZIL. Bahia, Mucuri, März [March] 1816, *Maximilian, Prinz zu Wied 53* (BR0000005575398! Fig. 14). Notes. Although Galeniacelosioides was synonymized with Microteamaypurensis (Moquin-Tandon 1849, Schmidt 1872, Walter 1909, Nowicke 1968) or M.debilis (Steudel 1841), the protologue (Sprengel 1818) contains the phrases “flosculi … brevissime pedicellati… styli duo” [flowers very shortly pedicellate, styles two], the characters found in M.celosioides. The description did not include reference to the presence of hooked outgrowths, a peculiar feature of M.maypurensis, and the short pedicels and two styles also exclude this species. Nevertheless, Moquin-Tandon (1849) reported his former intention to transfer Sprengel’s name to Microtea, apparently on the basis of his analysis of its protologue. He changed his mind probably because he did not examine the original material of the name. Galeniacelosioides was introduced anonymously to the Berlin Botanical Garden, from which it was described as new to science by the end of 1818 (Sprengel 1818). Although the source of introduction was not recorded, among European plant collectors in Brazil it was only Alexander Philipp Maximilian, Prince of Wied-Neuwied (1782–1867), who collected specimens early enough to bring the material to Berlin before the protologue of G.celosioides had been published. Prince Maximilian travelled in Brazil during 1815–1817, in the States of Rio de Janeiro, Bahia and Espírito Santo, mostly along the coast. He left for Europe in May 1817 and arrived at Lisbon on 1^st^ July. Very shortly after the arrival he started to distribute dried specimens, seeds and even living collections which he donated to botanical experts and gardens in Germany and Belgium. Large amounts of seeds were sent to Ghent, Antwerp and Enghien, but it is unknown which seeds were sent to Germany (Moraes 2009). A herbarium voucher of M.paniculata numbered 53 by C.G.D. Nees von Esenbeck, a German botanist who received a large set of specimens from Prince Maximilian for collaborative work, can be found in the Nees herbarium at BR. This number was cited by Walter (1909) as referable to M.paniculata (specimen destroyed at B). Along with the characters stated in the protologue of G.celosioides, this fact provides indirect evidence that Prince Maximilian’s collections were the likely source of the introduction of G.celosioides to the Botanical Garden in Berlin. There are no specimens of the original material of G.celosioides in existence, which probably was acquired by B and then destroyed (Stafleu and Cowan 1985). Since the characters of the Prince Maximilian’s specimen at BR are in good agreement with the protologue of G.celosioides, we designate this specimen as a neotype of the species name. This name is therefore the earliest one available for the species also known as M.paniculata.
M.paniculata Moq. in DC., Prodr. 13(2): 18 (1849), **syn. nov.** Lectotype (designated by Nowicke 1968: 350): BRAZIL. Bahia, “Serra Jacobina” [Villa do Barra, according to Moquin-Tandon (1849)], [1837], Blanchet 2709 (K000601204! isolectotype P00743956!).
M.longebracteata H.Walter, Pflanzenr. (Engler) 39: 129 (1909), **syn. nov**. Lectotype (Sennikov & Sukhorukov, designated here): BRAZIL. Prov. Bahia, zwischen den Campos und Vittoria, [1815], *Sellow 359* (B 10 0250568! isolectotypes – B 10 0250569! B 10 0250570!). Note: The main character distinguishing M.longebracteata from M.paniculata is the length of the bracts that is equal to those of the flowers (Walter 1909). However, the length of the bracts in both taxa is equal to that of the flower buds, and the length of fully opened flowers is greater than the length of the subtending bract.

###### Description.

Annuals or biennials; stems erect, up to 100 cm; leaves petiolate (petioles up to 2.0 cm), blades 3.0–10.0(12.0) cm long, 0.2–2.0 cm wide, cuneate, lanceolate to oblong, rarely ovate, glabrous or their margins and mid-rib below covered with papillae; inflorescence a spike, long and spreading, whip-like; flowers with a bract and two filiform bracteoles (often not well-visible), bracts longer than flowers at the beginning, then equal to the perianth segments; pedicels up to 1.0 mm at fruiting, perianth segments 5, oblong or ovoid; stamens 5–8; stigmas 2, thick; fruit with scattered short outgrowths, fruit body (1.0)1.1–1.4 × 0.9–1.1 mm, 1.5–2 times as long as the perianth (Fig. 4E, F); pericarp readily scraped off the seed; seed ~1.0 mm, with rough surface (Fig. 4H).

###### Habitat.

Forest margins, roadsides, river banks, on sandy and rocky substrates; altitudes up to 1000 m a.s.l.

###### Distribution.

**Figure 15.**
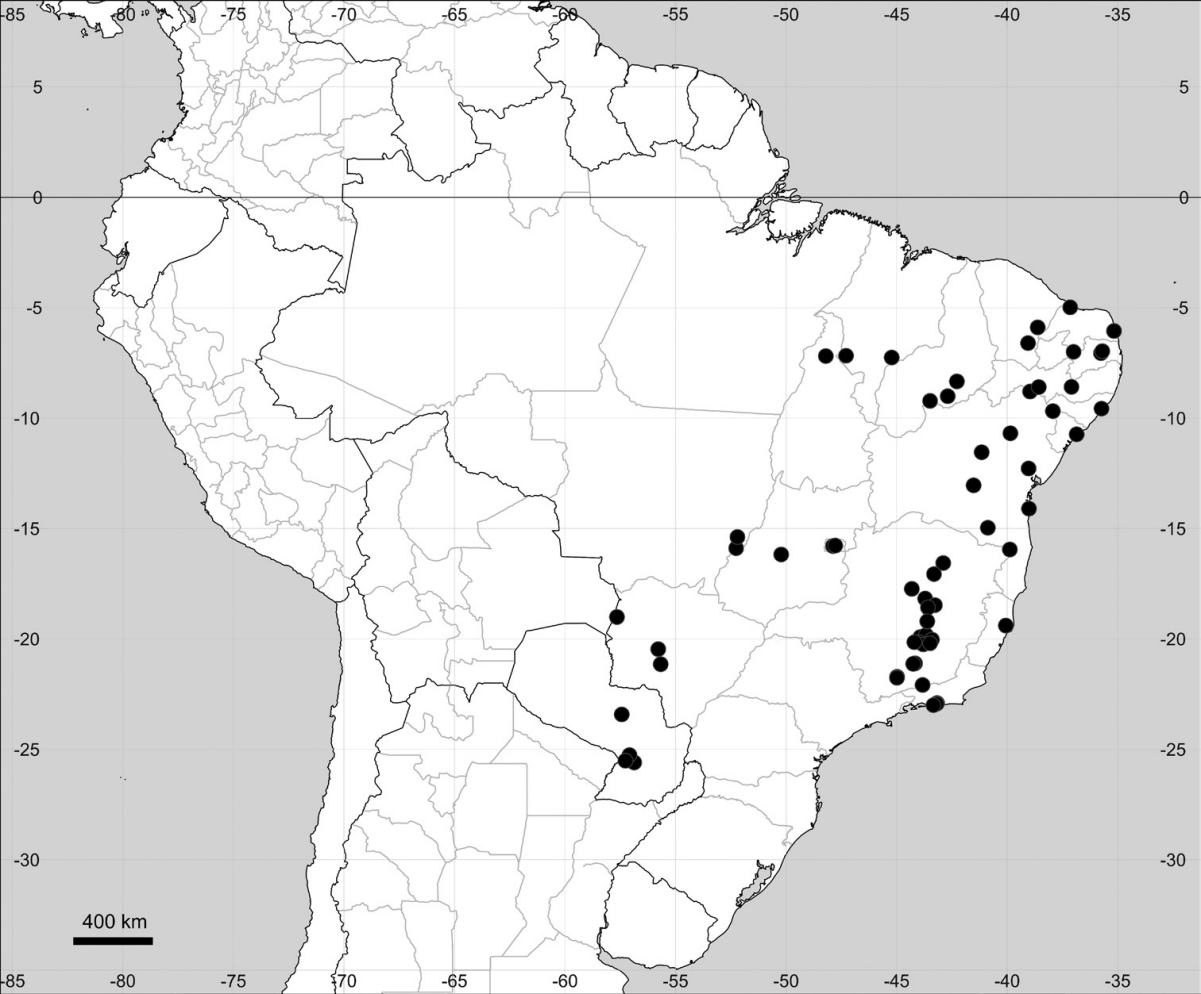
Distribution map of *Microteacelosioides*.

(Fig. 15) Eastern South America.

###### Specimens examined.

**BRAZIL** (selected specimens): **Alagoas**: Maceió, 4 Sep 1987, *S. Tsugaru & Y. Sano B-1470* (NYBG01014516 – image!); **Bahia**: Gloria Mun., Barra do Tarrachil, 17 Jul 1962, *G. Eiten & L.T. Eiten 4966* (K); Victoria da Conquista, 17 Jan 1965, *E. Pereira & G. Pabst 34875* (M); Rio Cumbuca, Mucugê, 4 Feb 1974, *R.M. Harley 15971* (K, P04598066, U1473440); Serra de Itiuba, Itiuba, 19 Feb 1974, *R.M. Harley 16204*(P04598067, U1473439); Milagres, 6 Mar 1977, *R.M. Harley 19451* (U1473444); Morro do Chapéu Mun., 980 m, 1 Jun 1980, *R.M. Harley 22970* (E, K, U1473442); Cachoeira do ferro Doido, Morro do Chapéu, 30 Nov 1980, *A. Furlan & al. s.n.* (PACA 76292); **Ceará**: Cedro, May 1933, *Ph. von Luetzelburg 26522* (M); Jaguaribe Mun., Maciço do Pereiro, 12 Apr 2011, *A.M. Miranda & K. Manso 6327* (HUEFS178925 – image!); **Distr. Federal**: Brasilia, 27 Oct 1965, *H.S. Irwin et al. 9600* (LE); Paranoá, 13 Dec 1965, *H.S. Irwin & al. 11258* (IAN122990 – image!); **Espírito Santo**: Linhares, 20 Apr 2011, *J. Meirelles et al. 624* (HUEFS193997 – image!); **Goiás**: Serra Dourada, 19 Jan 1966, *H.S. Irwin & al. 11812* (NYBG00864091 – image!); **Maranhão**: Loreto Mun., Loreto city, 11 Feb 1970, *G. Eiten & L.T. Eiten 10581* (K); Carolina Mun., Rodovia, 24 Apr 2008, *G. Pereira-Silva et al. 13260* (CEN00091428 – image!); **Mato Grosso**: Barra do Garças, 17 Jun 1966, *D.R. Hunt 6050* (K); Vale dos Sonhos, Barra do Garças Mun., 93 km S of Xavantina, 9 Nov 1968, *R.M. Harley & al. 10980* (K); **Mato Grosso do Sul**: Estrada Forte Coimbra, Lagoa do Jacadigo, Corumbá, 1 May 1989, *A. Pott et al. 4744* (PACA); Faz. Retirinho, Aquiduana, 23 Nov 1989, *A. Pott et al. 5433* (PACA); Maracaju Mun., Serra de Maracaju, 12 Nov 1993, *G. Hatschbach et al. 58937* (H1679509, K); **Minas Gerais**: Serra de Belo Horizonte, Belo Horizonte, 10 Feb 1927, *W. Hoehne s.n.* (PACA 76836); Diamantina, 3 Jun 1955, *E. Pereira 1680* (PACA); Estrada Diamantina–Milho Verde, 33 km from Diamantina, 3 Dec 1981, *N. Hensold et al*. *s.n.* (PACA 76282); Caeté Mun., Serra da Piedade, 10 Jan 1982, *N. Hensold et al. s.n.* (PACA 76294); Subida ao Pico do Itambé, Santo Antonio do Itambé, 5 Apr 1982, *A. Furlan et al. s.n.* (PACA 76296); Tiradentes, 6 Jun 1984 (PACA 76835); São Thomé das Letras, 30 Oct 1984, *I. Cordeiro et al. s.n.* (PACA 76291); Serra do Cabral, Joaquim Felício, 21 Nov 1984, *R.M. Harley et al. s.n.* (PACA 76295); N of Grão Mogol, 27 Nov 1984, *R.M. Harley et al. s.n.* (PACA 76283); Estrada Serra-Diamantina, Trinta Réis, 27 Jan 1986, *N.L. Monezes et al. s.n.* (PACA 76284); Itacambira, Rodovia to Juramento, 14 Feb 1988, *J.R. Pirani et al. 2272* (PACA); Caeté Mun., Morro da Piedade, Serra da Moenda, 24 Apr 1990, *J.A. Paula & S.B. Velten s.n.* (PACA 76847); Pico de Itabirito, Itabirito, 21 May 1994, *W.A. Teixeira s.n.* (PACA 76841); Retiro das Pedras, Brumadinho, 14 Dec 1998, *J. R. Stehmann & C.E.S. Ferreira 2399* (PACA); Faz. Santana, Salto da Divisa, 21 Aug 2003, *J.A. Lombardi et al. 5333* (PACA); Vilarejo do Funil, Rio Preto, 21 May 2004, *F.R.G. Salimena et al. 1284* (PACA); Parque Estadual de Grão Mogol, Grão Mogol, 13 Jun 2006, *C.V. Vidal 187* (PACA); Lima Duarte, 20 Nov 2006, *F.M. Ferreira et al. 1139* (K); Santana do Riacho Mun., Cachoeira Véu de Noiva, 15 Mar 2007, *M.S. Marchioretto 353* (PACA); Serra de Antônio Pereira, Samarco, Ouro Preto, 4 May 2007, *M. Messias et al. 1296* (PACA); Serra do Lenheiro, São João Del Rei, 25 Dec 2012, *M. Sobral 15294* (PACA); **Paraíba**: Paraiba do Norte, Serra Borborema, 20 Mar 1913, *Ph. v. Luetzelburg 12490* (M); Varzea, 23 Mar 1936, *Luetzelburg 27000* (M); Areia, 17 Jun 1953, *J.C. de Moraes 973* (IAN082599 – image!); Alagoa Nova Mun., Brejo Paraibano Reg., 5 Mar 2012, *E. Melo et al. 10917* (PACA); **Pernambuco**: Floresta, 26 Aug 1994, *M. Sales 319* (K); Buíque, 20 May 1995, *K. Andrade et al. 62* (K); **Piaui**: São Raimundo Nonato, 1 May 1978, *E. Laure 195* (P06806987); Serra Branca, São Raimundo Nonato, 7 Feb 1986, *L. Emperaire 2817* (P05197106); São Joao do Piaui Mun., Porfirio, Aug 1995, *F.G. Alcoforado Folho 480* (K); Caracol, 25 Feb 2011, *Melo et al. 9216* (PACA 115982) as *M.longebracteata*; Caracol Mun., Serra das Confusões, Serra Grande, 18 Jul 2011, *A.A. Conceição et al. 4037* (PACA); **Rio de Janeiro**: Rio de Janeiro [city], 1879, *A. Glaziou 11440* (LE); Guanabara, 7 Feb 1964, *W. Hoehne 5581* (P05197105); Corcovado, 5 Feb 1940, *B. Rambo 3581* (PACA); Próximo de Recreio dos Bandeirantes, Guanabara, 4 Apr 1964, *W. Hoehne 5708* (PACA); Est. da Guanabara, Pedra de Estauna, 31 Jan 1965, *Newton Santos 5409* (M); **Rio Grande do Norte**: Grossos, Salina Salmar, 27 Jun 2007, *A.A. Roque 137* (UFRN00004903 – image!); Nísia Floresta, Sete Lagoas, 18 Aug 2016, *V.F. Sousa 456* (UFRN00022373 – image!); **Sergipe**: Canindé de São Francisco, 9 Sep 2014, *K.M. Pimenta 589* (RB00956693 – image!); Pirambú Mun., [without date] *M. Ramos & E. Santos 98* (ASE0017438 – image!); **Tocantins**: Goias, Araguaina, 9 Mar 1982, *A. Krapovickas et al. 37841* (G); Rodovia, 1 Feb 2012, *R.M. Harley et al. 56656* (K); **PARAGUAY**: Valenzuela, 18 Mar 1884, *B. Balansa 4571* (B, P04598095); Tobati, [without date] *E. Hassler 3981* (P03321381); Dept. Cordillera, Cordillera de Altos, 6 Mar 1984, *A. Schinini 23957* (G); Dept. Cordillera, Tobati, 8 Feb 1991, *E. Zardini & C. Velázquez 26244* (B); Paraguay, Concepcion, Paso Horqueta, 18 Nov 1993, *E. Zardini & T. Tilleria 37460* (MW0581802).

##### 3. M. papillosa

Taxon classificationPlantaeCaryophyllalesMicroteaceae

M.S.Marchioretto & J.C.de Siqueira, Pesquisas, Botânica 48: 30 (1998).

###### Holotype.

BRAZIL. Minas Gerais, Estrada Conselheiro Mata, a 2 km do asfalto, Diamantina, 11 April 1982, *L. Rossi, A. Furlan, N.L. Menezes, N. Hensold, H.L. Wagner & E.M. Isejima 3317* (PACA!).

###### Description.

Perennial with a taproot or rarely annual (?) herb with caudex; stems erect, densely covered with short papillae; leaves papillate, oblong or lanceolate, appressed or somewhat spreading, cuneate, 2.0–4.0 cm long and 0.2–0.7 cm wide; inflorescence a spike; flowers subsessile, with the pedicels 0.25–0.5 mm long, with a bract and two bracteoles; perianth segments 5, oblong, greenish; stamens 6–8; stigmas 2, thick; fruit 1.1–1.25 × 0.9–1.1 mm, with short finger-shaped outgrowths (Fig. 5A, B); seed 0.9–1.0 mm, with rough surface (Fig. 5D). Morphologically, this species is most similar to *M.celosioides* and is distinguished by the papillate stems and leaves.

###### Habitat.

Sandy substrates at altitudes 500–1400 m a.s.l.

###### Distribution.

**Figure 16.**
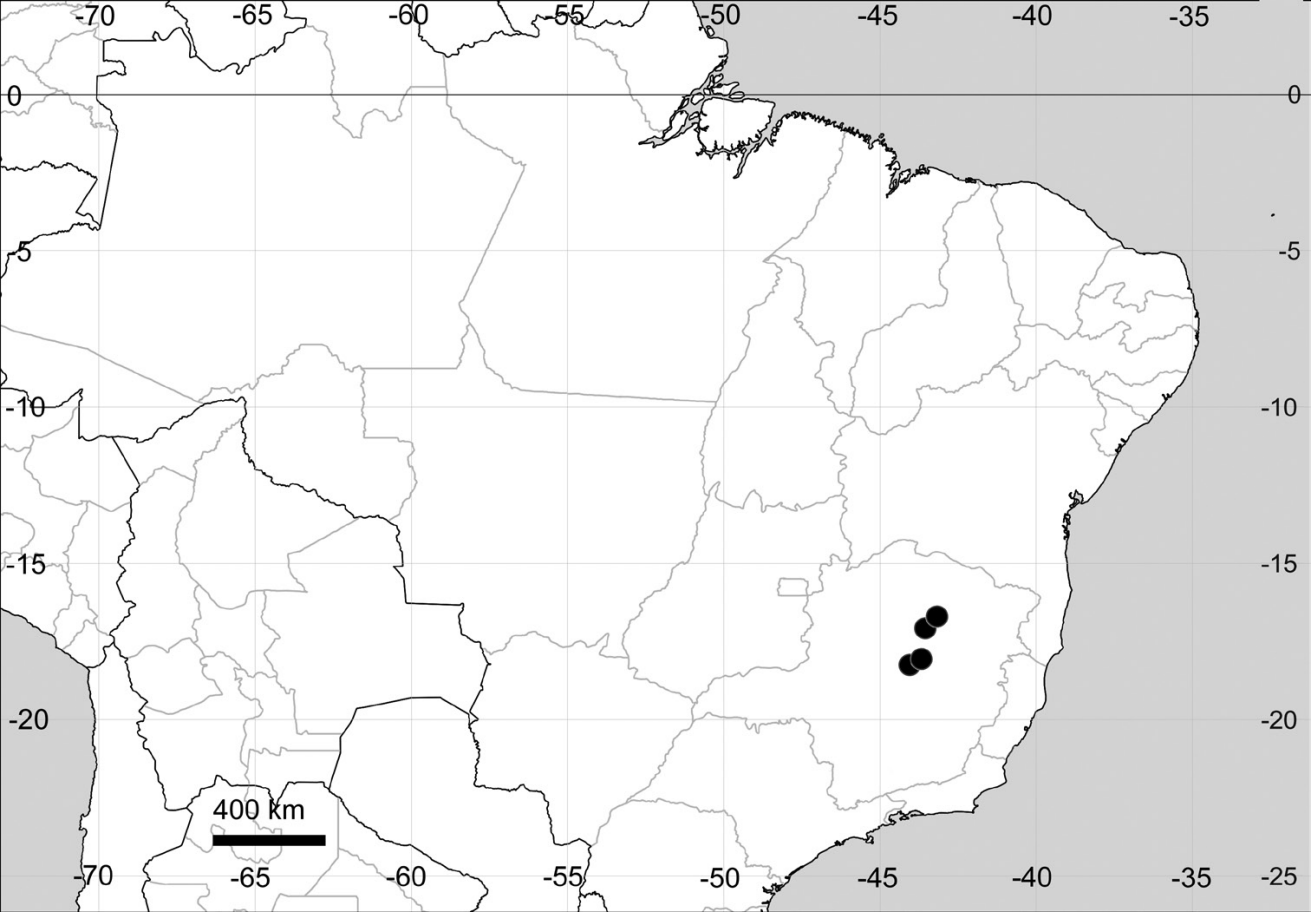
Distribution map of *Microteapapillosa*.

(Fig. 16) Endemic to Minas Gerais (Diamantina Mun.), Brazil.

###### Specimens examined.

**BRAZIL**. **Minas Gerais**: Diamantina Mun., Estrada Conselheiro Mata, a 2 km do asfalto, 11 Apr 1982, *L. Rossi et al. 3322* (PACA); N of Grão Mogol, 900–1000 m, 27 Nov 1984, *R.M. Harley et al. 36121 & 37090* (K); 15 km from Diamantina towards Mendanha, 2 Dec 1984, *B. Stannard et al. 36280* (K); Itacambira, 1220 m, 11 Nov 1988, *J.R. Pirani et al. 2272* (K); 5 km W de Diamantina, 1200 m, 16 Feb 1991, *M.M. Arbo et al. 5220* (K); Diamantina Mun., 14 km from Diamantina, 30 Jan 2000, *R.C. Forzza & R. Mello-Silva 1486* (G, K).

##### 4. M. scabrida

Taxon classificationPlantaeCaryophyllalesMicroteaceae

Urb., Ber. Deutsch. Bot. Ges. 3: 325 (1885).

M.paniculataMoq.var.scabrida (Urb.) Kuntze, Revis. Gen. Pl. 3(3): 268 (1898). Lectotype (Sennikov & Sukhorukov, designated here): BRAZIL. [Without exact location and date] F. Sellow s.n. (F0BN005735, image!). Note: The lectotype is chosen according to the protologue (Urban 1885). Urban’s collections of M.scabrida in B have probably been missing since 1945.
M.paniculatavar.latifolia Kuntze, Revis. Gen. Pl. 3(3): 268 (1898). Lectotype (Sennikov & Sukhorukov, designated here): BOLIVIA. Rio Yapacani, June 1892, O. Kuntze s.n. (B!).
M.foliosa Chodat, Bull. Herb. Boissier, ser. 2, 3: 418 (1903). Lectotype (designated by Nowicke 1968: 351): PARAGUAY. In regione collium “Cerros de Tobaty”, September 1900, *E. Hassler 6254* (MO216419, image! isolectotypes – P00743942! K000601209!). Note: This species was synonymized with M.paniculata (=M.celosioides) by Marchioretto and de Siqueira (1998). We have seen the original specimens cited in the protologue (Chodat and Hassler 1903): the specimens with the numbers 6254 (MO216419 – image! K000601209! P00743942!), 1649 (P03321197), 1988 (P00743946!) and 1988b (B! P00743944!) are M.scabrida, and the specimens with the number 7605 (K000601208! P00743941!) belong to M.sulcicaulis, a species described in the same article by Chodat and Hassler (1903). The authors mentioned in the publication three Microtea species: M.foliosa, M.paniculata andM.sulcicaulis (Chodat and Hassler 1903), andM.scabrida was omitted in the species list. The protologue of M.foliosa combines the characters of both M.scabrida andM.sulcicaulis, but the epithet “foliosa” belongs to M.scabrida with inflorescence leafy in its lower part. Based on the typification made by Nowicke (1968), and in agreement with Walter (1909), we synonymize M.foliosa with M.scabrida.
M.scandens Rusby, Mem. New York Bot. Gard. 7: 239 (1927). Holotype: BOLIVIA. La Paz, Iturralde, Ixiamas, alt. 244 m, 18 Dec 1921, *M. Cárdenas 1942* (NYBG01163848, image!).

###### Description.

**Figure 17.**
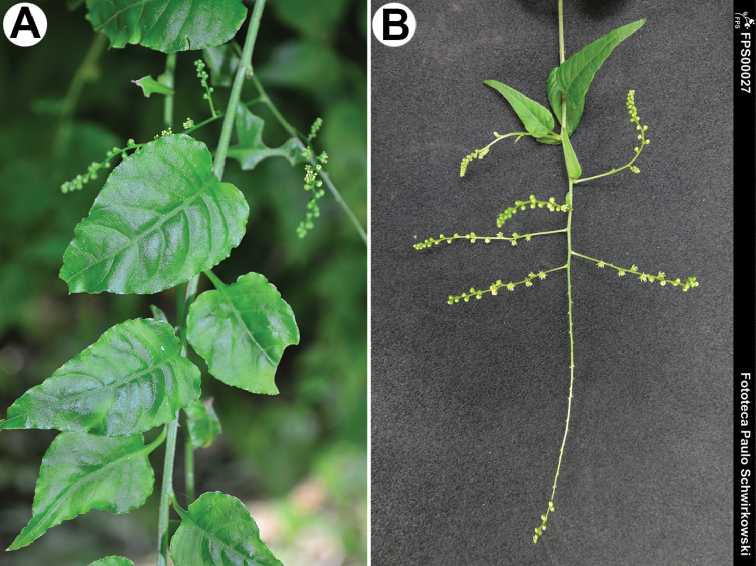
*Microteascabrida*: **A** a fragment of the shoot (São Bento do Sul, Santa Catarina, Brazil, 23 Sep 2016) **B** inflorescence (São Bento do Sul, Santa Catarina, Brazil, 14 Dec 2013). Photographs by Paulo Schwirkowski.

Leaning or twining perennial herb up to 150 cm, glabrous or slightly scabrid; leaves ovate or oblong (Fig. 17A), long-petiolate (petioles 1.0–4.0 cm), blades 5.0–10.0 cm long and 2.0–4.0 cm wide, basally truncate, apically acuminate; inflorescence lax, a spike (Fig. 17B); flowers almost sessile (pedicels up to 1.0 mm), with a bract and two bracteoles; perianth segments 5, whitish or green, oblong or ovoid, stamens 6–7, stigmas 2–3; fruit 1.75–2.0 mm long and 1.6 mm wide, with finger-shaped outgrowths, some of them basally concrescent (Fig. 5E, F); seed 1.3–1.5 mm, with verrucous surface (Fig. 5H).

###### Habitat.

Forests, shrub thickets; alt. up to 1000 m.

###### Distribution.

**Figure 18.**
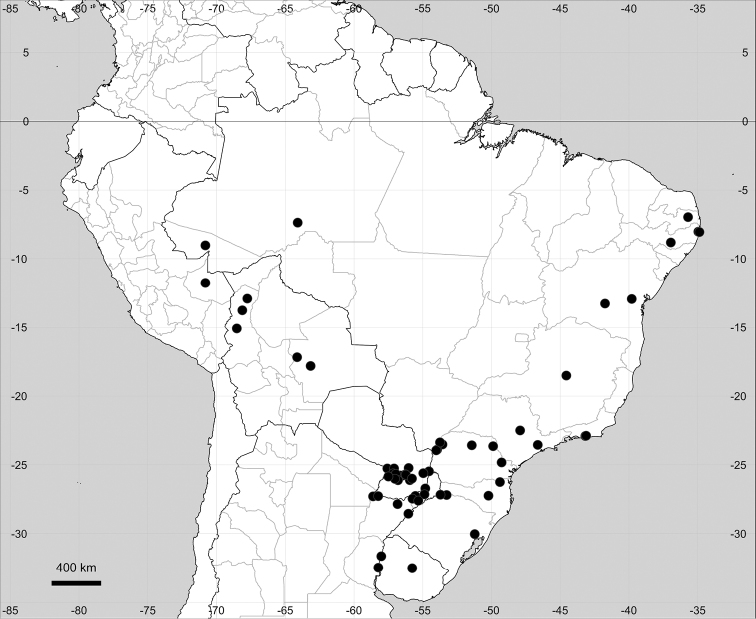
Distribution map of *Microteascabrida*.

(Sub)tropical parts of South America (Fig. 18).

###### Specimens examined.

**ARGENTINA**: **Chaco prov**.: Isla del Cerrito, 11 Dec 1971, *A.G. Schulz 17927* (G); **Corrientes prov**.: Itati, 6 Feb 1964, *T.M. Pedersen 68* (P04598111); Santo Tome Dep., Potrero Luna, 8 Dec 1981, *S.G. Tressens et al. 1612* (G); Ituzaingö Dept., Puerto Mora, 11 Dec 1973, *A. Krapovickas et al. 24329* (RB00272365 – image!); **Entre Rios prov**.: Concepción del Uruguay, Dec 1917, *L. Hauman s.n.* (BR); Dep. Uruguay, 31 Mar 1967, *T.M. Pedersen 8202* (E00621962, P04598112); Concordia Dept., Nueva Escócia, 24 Nov 1988, *N.M. Bacigalupo et al. 863* (B); **Misiones prov**.: Dept. San Ignacio, El Colorado, 24 Jun 1946, *G.J. Schwarz 2845* (L1678246); Dep. San Pedro, El Alcazar, 30 Mar 1949, *E. Schwindt 1428* (BR); Cainguás Dept., Oro Verde, 29 Apr 1949, *G.J. Schwarz 7789* (G); Candelaria Dept., Santa Ana, 10 Mar 1951, *J.E. Montes 15101* (P05197102); Leandro N. Alem Dept., Paso Carreta, 9 Mar 1969, *A. Krapovickas et al. 15003* (G); **BOLIVIA**: see lectotype of M.paniculatavar.latifolia (B); San Rafael, 27 Mar 1902, *R.S. Williams 222* (BM000019282, K); La Paz, Upper Rio Beni, Dec 1906, *J.W. Evans 53* (BM000019216); Santa Cruz Dept., Rio Yapacani, 8 Mar 1926, *J. Steinbach 7498* (BM000019275, E, K, U1473457); Prov. Sara, Dept. Santa Cruz, Rio Yapacani, 8 Mar 1926, *J. Steinbach 7498* (G); La Paz, Ixiamas, 18 Dec 1921, *M. Cárdenas 1942*(NYBG – image!); **BRAZIL**: **Acre**: Santa Rosa Mun., Rio Chambuiacu, 14 Mar 2002, *D.C. Daly et al. 11320* (NY00865147 – image!); **Amazonas**: Seringal São Francisco, Aug 1911, *E. Ule 9361* (G, K, L1678244); **Bahia**: [without exact location and year] *Blanchet s.n.* (LE); Rio Grongogy, Nov 1915, *H.M. Curran 148* (US01344703 – image!); Milagres, 6 Mar 1977, *R.M. Harley 19451* (E); Abaira Mun., 31 Jan 1992, *J.R. Pirani et al. 51372* (E00324053); **Minas Gerais**: [without exact location] 1816–1821, *A. Saint-Hilaire 43* (P04598127); **Paraná**: Tomazina, Barra Grande, 29 Jan 1911, *P. Dusén 11265* (K); Cerre Azul Mun., Ribeirao do Veado, 9 Feb 1960, *G. Hatschbach 6725* (L1678240); Foz do Iguaçu Mun., Parque Nacional, 18 Feb 1963, *G. Hatschbach 9737* (U1473447); Xambre Mun., 10 Dec 1965, *G. Hatschbach 13304* (B, P04598113); Icaraima & Porto Camargo, 20 Jan 1967, *G. Hatschbach et al. 4285* (U1473449); Icaraima Mun., Porto Camargo, 20 Jan 1967, *G. Hatschbach 15765* (B, P04598114); Altônia Mun., Porto Byington, 23 Jan 1967, *J.C. Lindeman & J.Y. de Haas 4396* (K); Cerro Azul Mun., Cabeceiras do Riberao do Tigre, 16 Dec 1992, *G. Hatschbach & O.S. Ribas 58457* (G); Cerro Azul Mun., Rua Serra da Paranapiacaba, 16 May 1997, *G. Hatschbach et al. 66536* (SJRP00009310 – image!); **Paraíba**: Areia, 15 May 1944, *J.M. Vasconcellos 240* (RB00272395 – image!); **Pernambuco**: [without exact location] 1838, *M. Gardner 1738* (G); Recife Mun., Caxangá, 29 Jul 1887, *Ridley et al. s.n.* (BM000019259); Recife, Mar 1936, *S. Vasconcellos 4097* (US01344756 – image!); **Rio de Janeiro**: [without exact location] 1816–1821, *A. Saint-Hilaire 376* (P04598132); Maricá Mun., Itaipuaçu, 27 Jan 1935, *Brade 29310* (B); Niterói, 6 Mar 1998, *M.C.F. dos Santos 153* (RB00766894 – image!); **Rio Grande do Sul**: [without exact location] 1816–1821, *A. Saint-Hilaire 2711* (P04598128); Iraí, Nov 1949, *K. Emrich s.n.* (PACA 48169); **Santa Catarina**: [without exact location] Jul 1840, *F. Müller 460* (K); Itapiranga ad fl. Uruguai superius, 16 Feb 1934, *B. Rambo 1788* (PACA), São Bento do Sul, Rio Natal, 14 Dec 2013, *P. Schwirkowski 128* (FURB36981 – image!); **São Paulo**: [without exact location] 25 Feb 1874, *A. Glaziou 2007* (P04598040); Serra de São Pedro, São Pedro, 22 Dec 1965, *J. Mattos & N. Mattos 13028* (PACA); **PARAGUAY**: **Asunción**: 13 Sep 1874, *B. Balansa 1988* (G, K, P04459470); **Caaguazú**: Caaguazú city, Nov 1874, *B. Balansa 1988b* (B, P00743944); **Caazapá**: San Juan Nepomuceno, 12 Dec 1989, *I. Basualdo 2794* (G); National Park Caaguazu, 24 Nov 1997, *E.M. Zardini & A. Benitez 47391* (P05197101); **Cordillera**: Tobati, 10 Jan 1903, *K. Fiebrig 677* (E, G, L, M); Tobati, 14 Jan 1903, *F. Fiebrig 737* (E, G, K, L1678243, LY, M) sub *M.foliosa*; Cerro Tobati, 28 Oct 1987, *R. Degun & E. Zardini 447* (G); Cerro Ybitu Silla, Tobati, 28 May 1988, *E. Zardini 4319* (G); **Guairá**: Villarica, 13 Sep 1874, *B. Balansa 1988* (P00743946); Villarica, 13 Nov 1945, *G.W. Teague 444* (BM000019276); Colonia Indepedencia, 30 Mar 1972, *T.M. Pedersen 10122* (K, P04598115); Tebicuary, 17 Nov 1978, *L. Bernardi 18685* (G); Melgarejo, 13 Mar 1989, *E. Zardini & C. Velásquez 11391* (E00047207, K); Cordillera de Ybytyruzu, 28 May 1989, *E. Zardini & U. Velásquez 12354* (G); **Paraguarí**: 15 Dec 1875, *B. Balansa 1988* (P04598096); prope Sapucay, 1885–1895, *E. Drake 1649* (P03321197); Sapucay, Jul 1913, *E. Hassler 11878* (BM000019235, E, G); La Rosada, 12 Dec 1979, *G. Schmeda 234* (G); Ybicui National Park, La Posada, 3 Feb 1992, *E. Zardini & P. Aquino 30230* (G); Estero del Ypoá, Trinchera Cué, 5 Aug 1993, *E.M. Zardini & T. Tilleria 36838* (B); **Alto Paraná**: [without exact location] 1909, *K. Fiebrig 5468* (BM000019222, E, G, L1678245, LY, M); **PERU**: reported for the Dept. Madre de Dios (Brako and Zarucchi 1993); **URUGUAY**: [without exact location and year] *J. Tweedie s.n.* (BM).

##### 5. M. sulcicaulis

Taxon classificationPlantaeCaryophyllalesMicroteaceae

Chodat, Bull. Herb. Boissier, ser. 2, 3: 419 (1903).

###### Lectotype.

(designated by Nowicke 1968: 351, first-step lectotype; Sennikov & Sukhorukov, second-step lectotype designated here): PARAGUAY. In regione fluminis Tapiraguay, Aug.[ust] [without year], *E. Hassler 4328* (K000601203! isolectotypes B! G! P00743937! P00634433!). Note: This species was synonymized with *M.celosioides* by Marchioretto and de Siqueira (1998, sub *M.paniculata*). We reinstate *M.sulcicaulis* to specific rank due to (1) strongly perennial life history, (2) stiff (not spreading) inflorescences, (3) larger fruit diameter, (4) alveolate seed surface (in *M.celosioides* it is rough but without alveolae), and (5) predominant distribution in subtropical South America (Paraguay, South Brazil, and SE Bolivia).

**Description.** Perennial herb with a taproot; stems up to 60 cm, sparsely branched, glabrous; rosulate leaves at least partially persistent, appressed to the stem, lanceolate to narrowly oblong, 3.0–8.0 × 0.5–1.0 cm, tapering into the short petiole; cauline leaves numerous, continuously decreasing in size towards the inflorescence, sessile; inflorescence a spike, branched, with lateral shoots directed upwards and not spreading or whip-like; pedicels up to 0.5 mm, flowers with a bract and two bracteoles; perianth segments 5, greenish with white margin or yellowish, ovoid; stamens 5–8; stigmas 2, thick; fruit 1.5–2.0 × 1.30–1.50 mm (Fig. 6A); pericarp readily scraped off the seed, with small finger-shaped outgrowths up to 0.3 mm (Fig. 6B); seed 1.3–1.5 mm, with alveolate surface (Fig. 6D).

###### Habitat.

Rocky and sandy substrates at altitudes up to 1500 m a.s.l.

###### Distribution.

**Figure 19.**
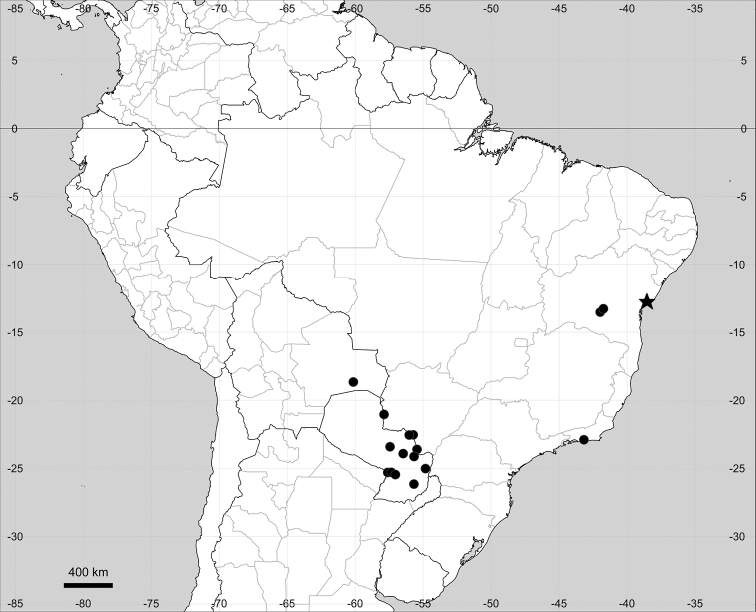
Distribution map of *Microteasulcicaulis* (circles) and *M.bahiensis* (star).

Subtropical South America (Fig. 19).

###### Specimens examined.

**BOLIVIA** (the first record for the country): **Santa Cruz Dept**., Fortin Suarez Arana, 19 Oct 1977, *C. Evrard 8203* (BR) as *Microtea* sp. Previously not reported for this country (Jørgensen et al. 2015); **BRAZIL: Bahia**: Rio de Contas Mun., Pico das Almas, 6 Nov 1988, *R.M. Harley et al. 25939* (K); Agua Quente Mun., Pico das Almas, 1400 m, 13 Dec 1988, *R.M. Harley & D.J.N. Hind 27232* (K); Mun. de Abaira, 13 Dec 1993, *W. Ganev 2615* (K); **Mato Grosso do Sul**: Pacuri, 12 Dec 1982, *G. Hatschbach 45918* (G); **Rio de Janeiro**: 1857, *A. Glaziou 16311* (BR); **PARAGUAY**: **Alto Paraguay Dept**.: Fuerte Olimpo, 24 Oct 1946, *T. Rojas 13637* (E); **Amambay Dept**.: Pedro Juan Caballero, Bela Vista, 23 Nov 1963, *J. Correa Gomes 1465* (G); Pedro Juan Caballero, 19 Oct 1986, *T.M. Pedersen 14669* (G); Estancia 5 Hermanos, 2 Nov 1997, *I. Basualdo 6437* (G); National Park Cerro Corá, Cerrado, 350 m, 10 Nov 1999, *E.M. Zardini & P. Baéz 52218* (P05197108); **Caazapá Dept**.: Tavai, 7 Dec 1988, *F. Mereles 2122* (G); **Canindeyú Dept**.: Nanduro Kai, 1 Nov 1978, *L. Bernandi 18347* (G); Lagunita, 23 Sep 1988, *T.N. Pedersen 15089* (G); [without exact location] 11 Oct 1996, *B. Jiménez & G. Marin 1616* (BM000527219); Mbaracayú Natural Reserve, 31 Oct 1998, *E.M. Zardini & I. Chaparro 49429* (B 10 0058099); **Central Dept**.: Ypacarai Lake, Dec 1913, *E. Hassler 12395* (BM000019236, E, G, L1678248, LY); **Concepción**.: nr Concepción, Oct 1901, *E. Hassler 7605* (G, K000601208, LY, sub *M.foliosa*); **Cordillera Dept**.: Piribebuy, 11 Jan 1877, *B. Balansa 2576* (P04598143); Colonia Rosado, 26 Oct 1986, *A. Schinini & E. Bordas 24850* (G); **San Pedro Dept**.: Yaguareté forest, 24 Aug 1995, *E. Zardini & A. Vargas 43638* (MW).

##### 6. M. bahiensis

Taxon classificationPlantaeCaryophyllalesMicroteaceae

M.S.Marchioretto & J.C.de Siqueira, Pesquisas, Botânica 48: 11 (1998).

###### Holotype.

BRAZIL. Estado Bahia, Munícipio de Salvador, ca. 30 km a N do Centro de Salvador, Estrada para o aeroporto, arredores de Itapuã, dunas [Bahia State, Salvador Municipality, ca. 30 km N from Salvador city, on the way to the airport, surroundings of Itapuã, dunes], 23 May 1981, *Carvalho, Mori & Boom 706* (CEPEC! isotypes – ALCB, NY).

###### Description.

Perennial herb or dwarf subshrub, glabrous, up to 30 cm tall; caudex well-developed; stems erect or ascending; rosulate leaves up to 9.0(10.0–12.0) cm, obovate or oblong, mostly persistent at fruiting; cauline leaves rhombic, ovate or obovate, cuneate and shorter (up to 2.0 cm) than the rosulate leaves; inflorescence a spike, mostly one-sided; flowers sessile or very shortly pedicellate (pedicels up to 0.5 mm); bracteoles present, very short, perianth segments 5, greenish with white margins, oblong or ovoid; stamens 8; stigmas 2, thick; fruit roundish, 1.1–1.3 × 1.0–1.2 mm, with short finger-shaped outgrowths (Fig. 6E, F); seed 1.1–1.3 mm, with rough surface (Fig. 6H). Note: We were unsuccessful in extracting DNA from the available specimens. However, the characters of this species indicate its position within the type subgenus.

###### Habitat.

Sand dunes at altitudes up to 500 m a.s.l.

###### Distribution.

Endemic to Bahia state, Brazil (Fig. 19).

###### Specimens examined.

**BRAZIL**. Bahia State, Salvador Mun., Itapuã, 27 Feb 1983, *P. de Queiroz 496* (ALCB); Itapuã, 20 Apr 1983, *P. de Queiroz 544* (HUEFS, PACA); Bahia [state], Salvador, Dunas de Itapuã, nr Hotel Stella Maris, N from Condomínio Alamedas da Praia, 8 Jun 1993, *P. de Queiroz 3211* (K, PACA).

##### 7. M. portoricensis

Taxon classificationPlantaeCaryophyllalesMicroteaceae

Urb., Ber. Deutsch. Bot. Ges. 3: 324 (1885).

###### Lectotype.

(designated by Nowicke 1968: 348, first-step lectotype; Sennikov & Sukhorukov, second-step lectotype designated here): PUERTO RICO. Cabo Rojo, in campis, 20 January 1885, *P. Sintenis 717* (S-R-3531 – image! isolectotypes P00743938! B 10 0296282! B 10 0296820! BM000019288! G! K000601210! P00743938! P00743939! L1678241! L1678242! LE!).

###### Description.

Annual, glabrous, stems decumbent, up to 30 cm; rosulate leaves up to 8.0 cm, long-petiolate (petioles up to 3.0 cm), obovate or oblong, mostly persistent at fruiting; cauline leaves rhombic or ovate, cuneate; inflorescence a spike, one-sided, flowers sessile or very shortly pedicellate (pedicels at fruiting ~1.0 mm); bracteoles mostly absent or tiny; perianth segments 4–5, greenish, lanceolate or oblong; stamens 4–5; stigmas 2, thick; fruit almost orbicular or broadly ovate, 0.9–1.1 × 0.9–1.0 mm, reticulate, without any projections (Fig. 7A, B); seed 0.9–1.0 mm, with rough surface (Fig. 7D). Note: This species is assigned here to the type subgenus, although it was not included in the molecular analysis. Morphologically, it is closely related to *M.debilis*.

###### Distribution.

**Figure 20.**
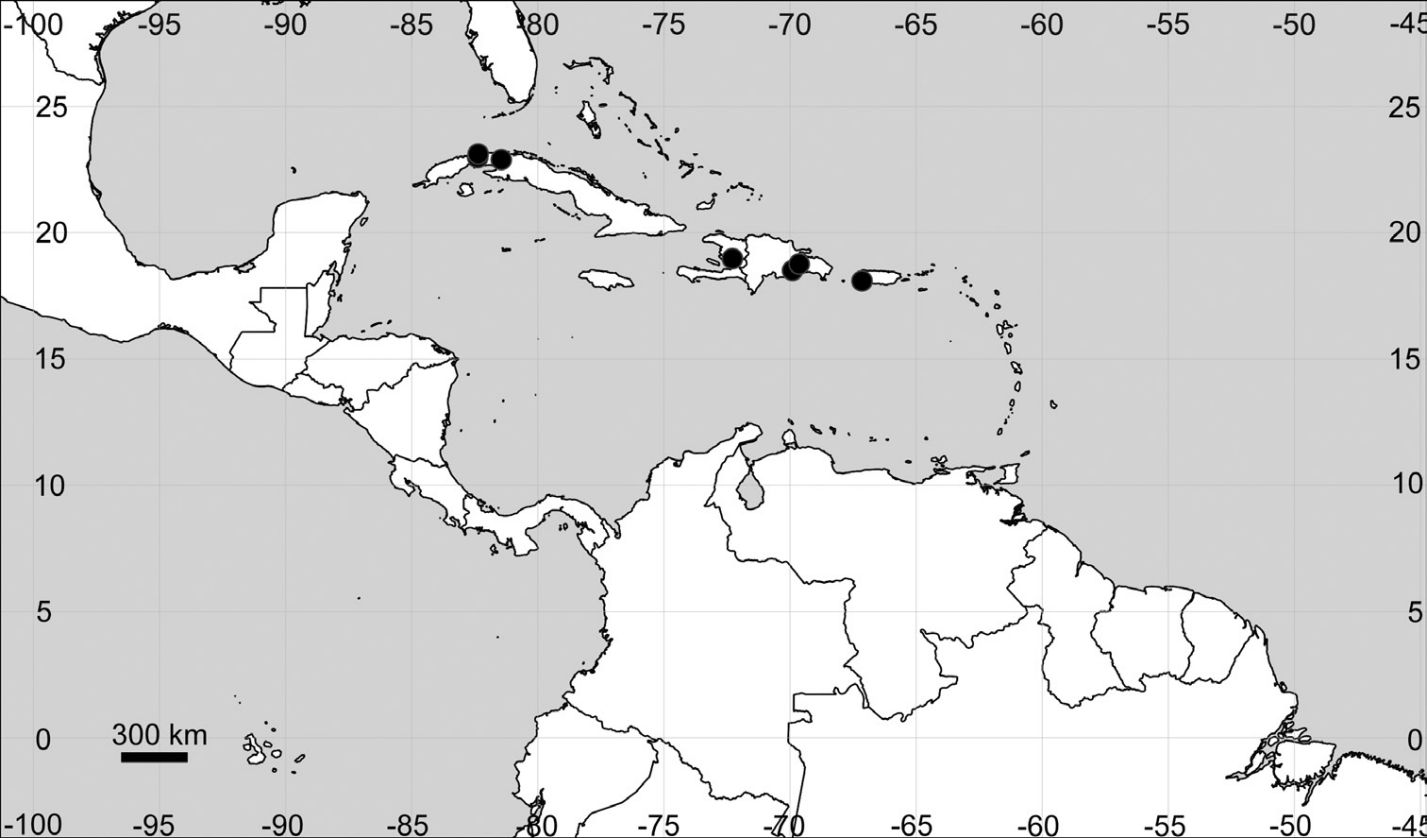
Distribution map of *Microteaportoricensis*.

Endemic to the Greater Antilles (Fig. 20).

###### Specimens examined.

**DOMINICAN REPUBLIC**: reported by Moscoso (1943) and Nowicke (1968 [“herbarium S”] (n.v.); **HAITI**: reported by Nowicke (1968), herbarium S (n.v.); **CUBA**: Santiago de las Vegas, 10 May 1904, *H.A. van Hermann 121* (BM00019279, P04598109); Ramón de la Sagra, [without date] *A. Jamain s.n.* (P04598110); Limonar, 1864, *Angel 771* (LE); Havana, 3 Nov 1921, *E.L. Ekman 13408* (G); **PUERTO RICO**: see type specimens; Cabo Rojo, 1864, *Grosourdy 13* (P04598159); Laguna Derrumadero, nr Bayaguana, 7 Sep 1981, *T. Zanoni & M. Mejia 16407* (NY01509884 – image!).

##### Microtea subgen. Ancistrocarpus

Taxon classificationPlantaeCaryophyllalesMicroteaceae

(Kunth) Sukhor. & Sennikov, comb. & stat. nov.

urn:lsid:ipni.org:names:77193077-1

Ancistrocarpus Kunth, Nov. Gen. Sp. [quarto] 2: 186 (1817). ≡Microteasubgen.Eumicrotea H.Walter, Pflanzenr. (Engler) 39: 127 (1909), nom. inval. (Art. 21.3). Type: M.maypurensis (Kunth) G.Don.

###### Description of the subgenus.

Annuals; bracteoles present; pedicels conspicuous (1.35–3.0 mm long); flowers single or clustered (2–6 per node); stigmas 3–5, thin. The species are mostly distributed in Brazil, with irradiations to the neighbouring countries.

##### 8. M. glochidiata

Taxon classificationPlantaeCaryophyllalesMicroteaceae

Moq. in DC., Prodr. 13(2): 18 (1849).

###### Lectotype.

(Sukhorukov & Sennikov, designated here): BRAZIL. Piauhy [Piauí], near Boa Esperança [-6.809768, -41.380520], February 1839, *Gardner 2311* (K000601202! isolectotypes B! P00743948! P00743949! P00743950!). Notes: The lectotype specimen, *Gardner 2311*, was collected when G. Gardner used the hospitality of Rev. Marcos de Araújo Costa (Gardner 1846), who was a land-owner, amateur botanist and educator in the province of Piauí, Brazil. The other collections cited in the protologue (Moquin-Tandon 1849), *Blanchet 2680* from Villa di Barra, belong to *M.maypurensis* (P00798999) and *M.celosioides* (BM000019298).

###### Description.

Annual, glabrous, up to 40 cm; stem erect, usually branched from the base with mostly persistent rosulate leaves; leaves linear to oblong, cuneate, 1.0–3.0 cm long, 0.1–0.4 cm wide; inflorescence thyrsoid, flowers 1–6 at each node, pedicels at fruiting 1.5–2.5(3.0) mm long; each flower with a bract and two bracteoles; perianth segments 5, white or yellowish, broadly ovoid or suborbicular, 0.8–1.0 mm long, imbricate; stamens 5–8; stigmas 3–5, filiform; fruit orbicular, its body 1.0–1.2 mm, covered with plumose outgrowths 0.4–0.7 mm long (Fig. 7E, F); pericarp easily scraped off the seed; seed ~1.0 mm, with slightly alveolate surface (Fig. 7H).

###### Habitat.

Forest margins, roadsides, ruderal sites, on sandy substrates at altitudes up to 500 m a.s.l.

###### Distribution.

**Figure 21.**
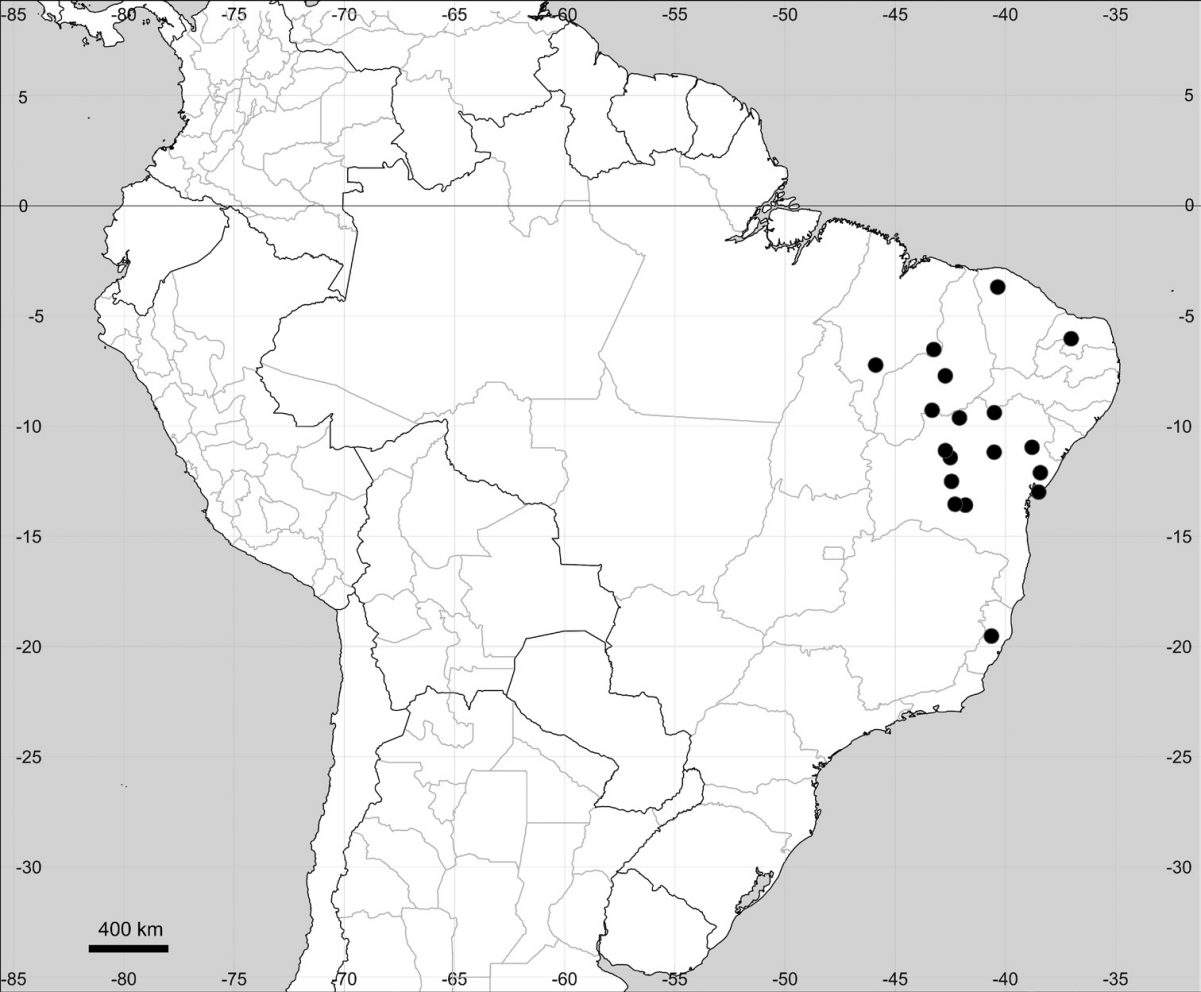
Distribution map of *Microteaglochidiata*.

(Fig. 21) Eastern tropical Brazil.

###### Specimens examined.

**BRAZIL: Bahia**: Jacobina, [without date] *Blanchet 2630* (P04598062); Nova da Rainha, [without date] *Martius 304*/*18* (M); Gentio do Ouro, 22 Feb 1977, *R.M. Harley 18907* (E, K, M, P05197098, U1473427); Gentio do Ouro Mun., nr Santo Inácio, 26 Feb 1977, *R.M. Harley 19120* (E, P05197099, U1473426); Upper São Francisco river, Caldeirão Grande, 500 m, 18 Apr 1980, *R.M. Harley 21511* (K); Riacho Grande, 4–5 km NE from Itatim, Santa Terezinha, 16 May 1983, *L. Noblick et al. 3243* (PACA); Tucano Mun., 20 Feb 1992, *A.M. de Carvalho & D.J.N. Hind 3841* (G, K, PACA); Paramirim Mun., Lago do Leito, 17 Jan 1997, *G. Hatschbach et al. 65893* (H1693402); Rio de Contas Mun., 5 Feb 1997, *M.L. Guedes et al. 5118* (K); Remanso, 27 Feb 2000, *M.R. Fonseca et al. 46314* (K, P000868555); Remanso, 10 Mar 2005, *L.P. Queiroz et al. 10070* (HUEFS093066 – image!); **Ceará**: Sobral, 8 Apr 1984, *A. Fernandes 12451* (ASE0023720 – image!); **Espírito Santo**: between Colatina & Patrimônio, 4 Dec 1971, *A.P. Duarte 13986* (PACA); **Maranhão**: Sao Raimundo das Mangabeiras Mun., 15 Mar 1962, *G. Eiten & L.T. Eiten 3674* (K); Barao do Grajau, 21 Jan 2012, *R.M. Harley et al. 56455* (K); **Pernambuco**: Petrolina, 22 Jan 1970, *P. Carauta 1008* (RB169035 – image!); **Piauí**: see lectotype and isolectotypes of *M.glochidiata*; Caracol, 25 Feb 2011, *E. Melo et al. 9216* (PACA); **Rio Grande do Norte**: Jucurutu, 1 Jun 2008, *A.A. Roque 570* (HUEFS164628 – image!).

##### 9. M. maypurensis

Taxon classificationPlantaeCaryophyllalesMicroteaceae

(Kunth) G.Don, Hort. Brit. [Loudon]: 98 (1830).

**Figure 22.**
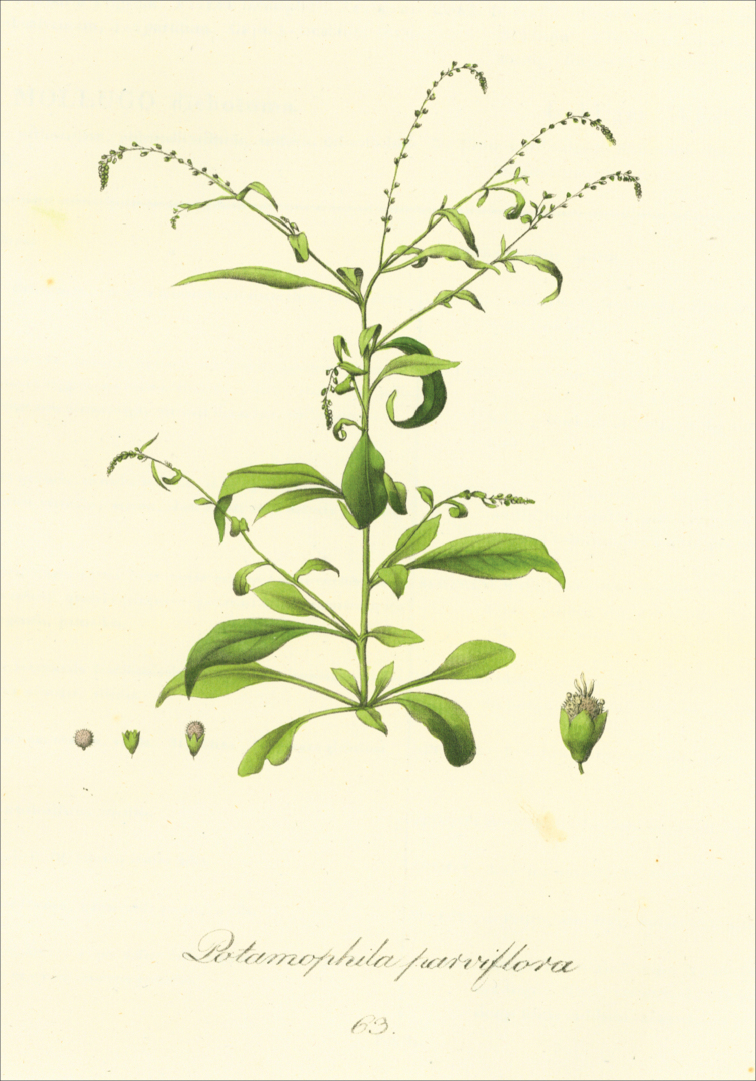
Lectotype of *Ancistrocarpusschrankii* (fig. 63 in Schrank, 1821). Image provided by the library of Biological Faculty, Lomonosov Moscow State University.

Ancistrocarpusmaypurensis Kunth, Nov. Gen. Sp. [quarto] 2: 186 (1817). Lectotype (Sennikov & Sukhorukov, designated here): COLOMBIA. “In pratis Maypure”, ex herb. Bonpland, ex herb. Kunth s.n. (B 10 0296283!). Note: Kunth, who revised the main set of Humboldt’s and Bonpland’s collections at P, acquired a large part of this herbarium. After his death, the collections were transferred to the Botanical Museum in Berlin (Urban 1881), where they partly survived.
Ceratococcamaypurensis Humb. & Bonpl. ex Roem. & Schult., Syst. Veg., ed. 15, 6: 800 (1820). Holotype: Colombia. Maypure, Cataracta, Orinoco, A.J.A. Bonpland & F.W.H.A. von Humboldt s.n. (B-W 06266-01!). Notes: Ceratococcamaypurensis was apparently based on a duplicate of the collection on which Ancistrocarpusmaypurensis was described earlier by Kunth. However, a specimen in the Herbarium of Willdenow was not accessible to Kunth at the time when the protologue was prepared (McVaugh 1955).
Potamophilaparviflora Schrank, Pl. Rar. Horti Monac. 2: tab. 63 (1821), nom. illegit., non R.Br. 1810.
Ancistrocarpusschrankii Ledeb., Index Seminum Horti Academici Dorpatensis 1821 (Appendix I): 21 (1821). Described on the basis of plants cultivated in the Botanical Garden in Munich. Lectotype (Sennikov & Sukhorukov, designated here): [icon] fig. 63 in Schrank (1821). (Fig. 22). Note: von Ledebour (1821) intended (Art. 6.11) to introduce his new species name as a replacement name for the later homonym published by Schrank, and both names are therefore necessarily homotypic. Schrank (1821) published his new species names based on seeds sent or brought from Brazil by C.F. von Martius. Original herbarium collections of Potamophilaparviflora Schrank were not found at M and most likely had never been prepared (H.-J. Esser, pers. comm. 2018), and the illustration published as part of the protologue is the only original element available for lectotypification (Art. 9.4). The protologue of Potamophilaparviflora indicates that the new species has five styles (although only three are visible in the accompanying illustration) and a pericarp with setae. Coupled with pedicellate flowers and petiolate leaves, easily recognizable in the illustration, these characters are indicative of Microteamaypurensis, of which P.parviflora (Ancistrocarpusschrankii) is a later synonym. This agrees with the conclusions of Moquin-Tandon (1849), Walter (1909) andNowicke (1968). Schrank (1821) stated that the new species was collected in “Brasilia prope sinum Omnium Sanctorum ad flumina locis umbrosis”. There is a gathering, Herb. Martius *2198* (M 0274659, M 0274661, M 0274662), which was collected by Martius in Bahia, Cachoeira, along the Paraguaçu River at the distance of ca. 35 km from Baía de Todos os Santos (Bay of All Saints), in December 1818 (see the route of Martius’ expedition in Tiefenbacher (1983)). This is the likely type locality of P.parviflora, and the gathering may be the voucher for the seed collection sent by Martius to the Munich Botanical Garden. The plants of this gathering are unmistakeably referable to M.maypurensis. Two other specimens, Herb. Martius 2309 (M 0274665, M 0274666), collected by Martius in Bahia, Monte Santo, in April 1819, were mistakingly labelled as P.parviflora. They clearly disagree with the protologue and belong to M.celosioides. This curatorial mislabelling probably occurred because of the confusingly similar localities on its label and in the protologue.
M.sprengelii Moq. in DC., Prodr. 13(2): 19 (1849). Neotype (Sennikov & Sukhorukov, designated here): BRAZIL. Bahia, Rio Belmonte, [Aug. 1816], *Maximilian, Prinz zu Wied 53* (BR000005537679, image!). Note: Moquin-Tandon (1849) based the protologue of Microteasprengelii entirely on the description of M.maypurensis in Sprengel (1820). He stressed the diagnostic character of opposite leaves, which is almost impossible in Microtea. However, Sprengel’s description closely matches the characters of M.maypurensis except for the opposite leaves, and unlikely belongs to any other species. Moquin-Tandon (1849) assumed a technical error in Sprengel (1820), with which we agree. Sprengel noted specifically that he based the description on plants from Brazil. No relevant specimens survived. As a neotype, we designate a specimen of M.maypurensis collected in Brazil by Prinz Maximilian, a contemporary collection that may have been available to Sprengel.
M.glochidiataf.lanceolata Chodat & Hassler, Bull. Herb. Boiss., ser. 2, 3: 418 (1903). Lectotype (Sennikov & Sukhorukov, designated here): Paraguay. Ad marginem silvae [prope] Caraguatay, Aug.[ust] [1897], *E. Hassler 3126* (P00743952! isolectotypes P00743951! P00743953!). Note: Our synonymy confirms the opinion of Heimerl (1912).

###### Description.

Annual or biennial, glabrous; stems erect, up to 60 cm, branched; rosulate leaves oblong, usually withered, lower leaves oblong or spatulate, cuneate, petiolate (petioles up to 2.5 cm), 3.0–8.0 cm long and (0.2–0.4)0.5–2.0 cm wide (sometimes narrower), acuminate; inflorescences a spike, not one-sided, often spreading; flowers solitary (rarely two per node), with a bract and two bracteoles, pedicellate (pedicels 1.5–3.0 mm), perianth segments 5, oblong to ovoid, white or yellowish; stamens 5–8, stigmas 3–5, filiform; fruit slightly protruding from the perianth or up to twice its length, fruit body 1.0–1.1 mm across, with outgrowths 0.2–0.5 mm long terminating in a group of 2–4 hooked hairs (Fig. 8A, B); pericarp readily scraped off the seed; seed ~1mm, with rough surface (Fig. 8D).

###### Habitat.

Forests or ruderal sites; 0–1500 m.

###### Distribution.

**Figure 23.**
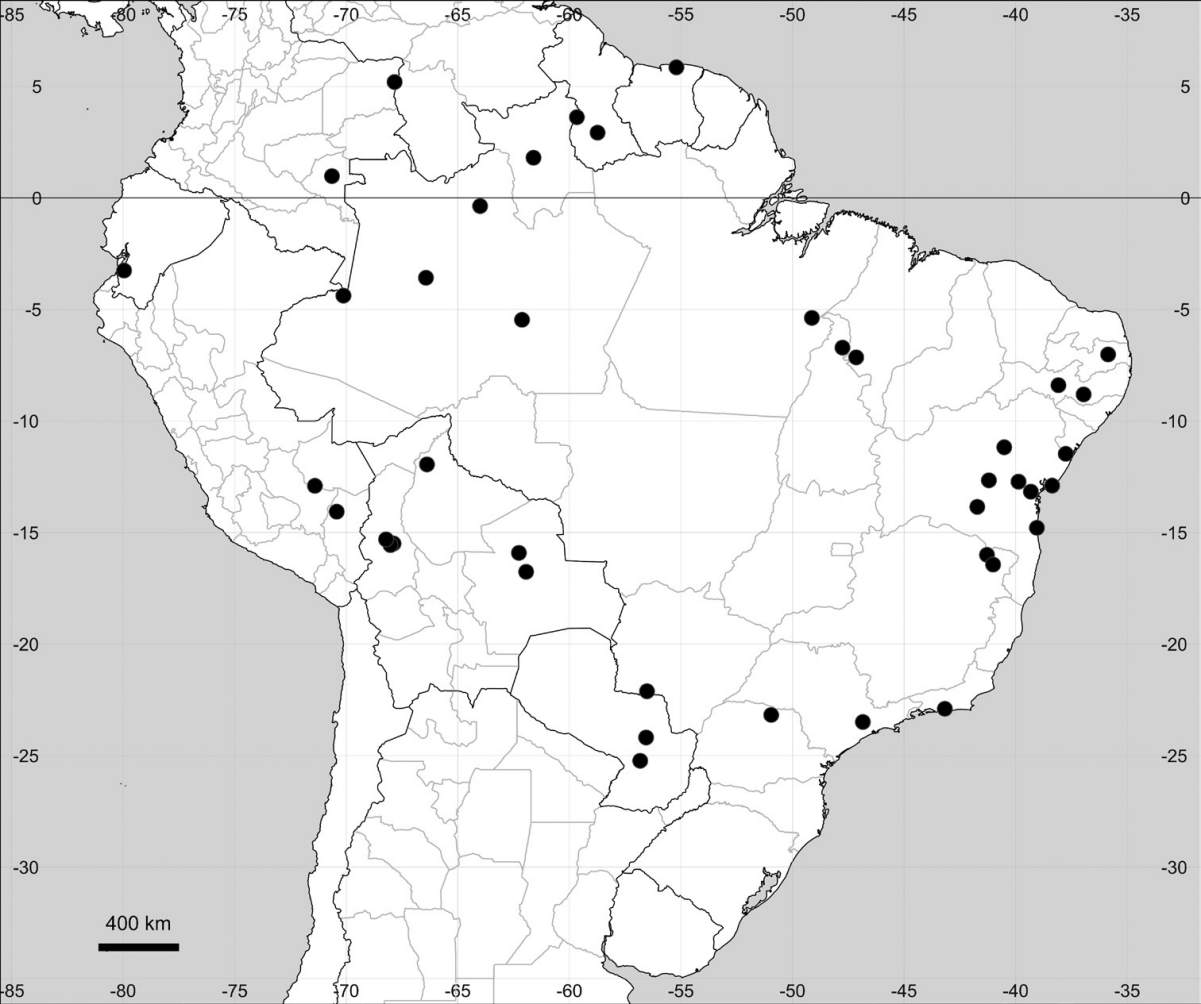
Distribution map of *Microteamaypurensis* in its native range.

Tropical South America (Fig. 23).

###### Specimens examined.

**Figure 24.**
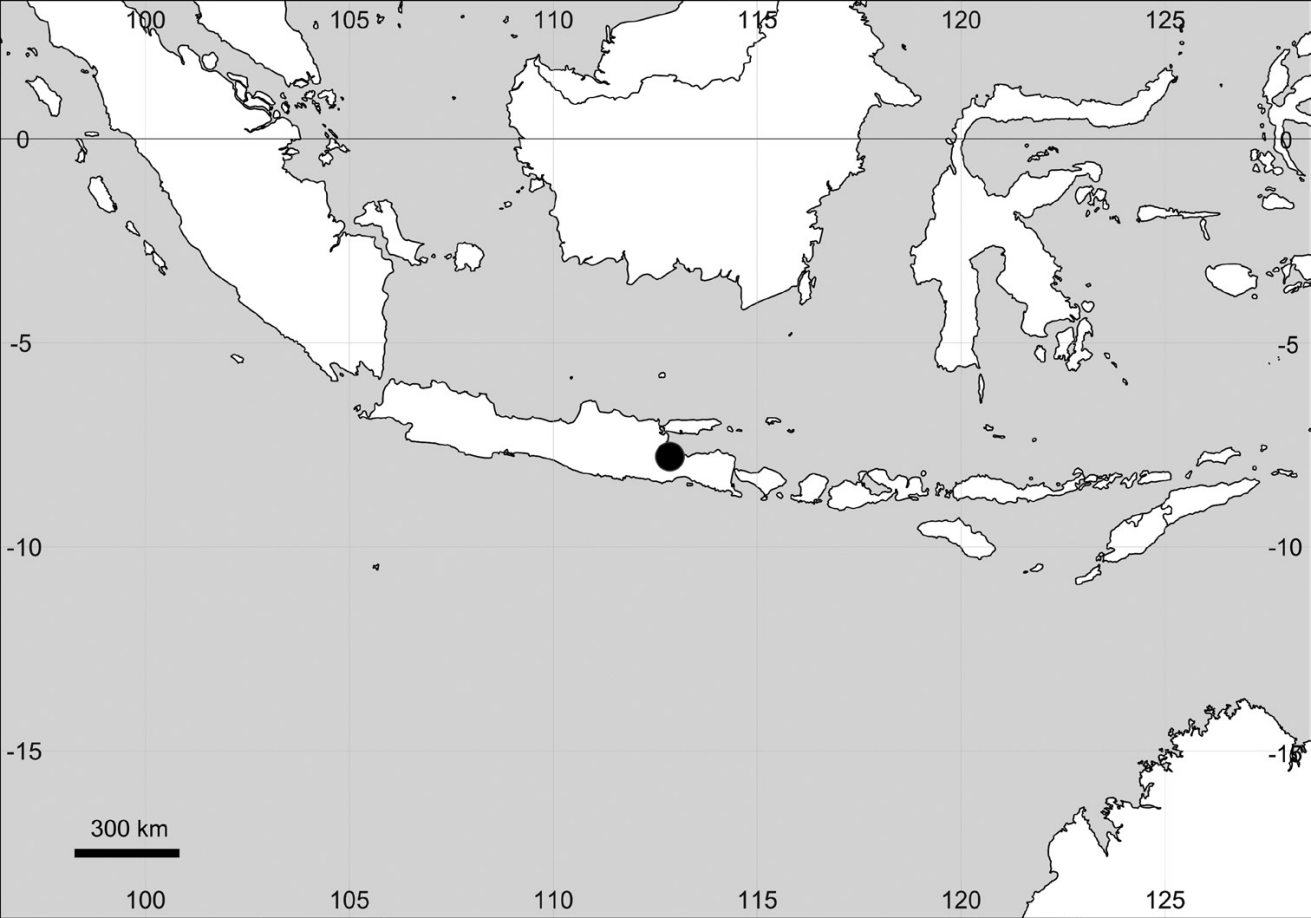
The record of *Microteamaypurensis* as an alien plant in Indonesia.

**BOLIVIA**: **La Paz Dept**.: Beni river, Jul 1886, *H.H. Rusby 1379* (BM000019263, E, G, LE, P04598104); nr Guanay, 1892, *M. Bang 1589* (B, BM000019206, E, G, K); Tipuani, Hacienda Simaco, 1400 m, Jan 1920, *O. Buchtien 5404* (K, M); Tipuani, 1400 m, 29 Mar 1923, *O. Buchtien 7290* (E, G); Mapiri, 850 m, 29 Nov 1926, *O. Buchtien 694* (M); **Santa Cruz Dept**.: Ñuflo de Chávez prov., Pascana Ministro, 12 Jun 1995, *J.R. Abbott 16976* (K); Ñuflo de Chávez prov., Lomerio, 14 Apr 1995, *F. Mamani & M. Saucedo 817* (MEXU); **BRAZIL: Amazonas**: Rio Juruá, Juruá Miry, May 1901, *E. Ule 5503* (G, L1678235); Rio Branco, Nov 1913, *J.G. Kuhlmann 120* (RB00272533 – image!); Rio Negro between Ilha Uabetuba & Ilha da Silva, 14 Oct 1971, *G.T. Prance et al. 15235* (NY00779040 – image!); Rio Jutai, 17 Nov 1975, *N.A. Rosa & L. Coelho 563* (IAN151165 – image!); **Bahia**: [without exact location] 1834, *Blanchet s.n.* [herb. De Candolle] (G00687542); Jacobina, [without date] *Blanchet 2588* (P00743954, a plant in the middle]; Andaraí, 500–600 m, 13 Feb 1977, *R.M. Harley 18632* (E, K, P05197100); Iaçu Mun., Lagedo Alto, 25 Sep 1984, *L.R. Noblick & M.J. Lemos 3407* (PACA); Ilheus Mun., Rodovia, 10 Apr 1986, *J.L. Hage s.n.* (G); Dom Basilio Mun., 28 Dec 1989, *A. de Carvalho et al. 2679* (G, PACA); Jacobina Mun., 30 Nov 1992, *M.M. Arbo et al. 5450* (K); Iaçu Mun., Rio Paraguacu, 10 Apr 1992, *G. Hatschbach et al. 56968* (G, K); Rio Jecuriçá, 16 Jan 1997, *M.M. Arbo et al. 7276* (G, K); **Maranhão**: Carolina Mun., National Park Chapada das Mesas, 9 Apr 2016, *A.C. Sevilha et al. 5723* (CEN00097885 – image!); **Minas Gerais**: Pedra Azul, 12 Dec 1984, *A.M. Giulietti et al. 36297* (K); towards Jequitinhonha, Pedra Azul, 20 Oct 1988, *R.M. Harley et al. 25232* (K, PACA); **Pará**: Marabá to Altamira, 16 Jan 1976, *P. Bamps 5170* (BR); **Paraíba**: Esperança, 14 Sep 1958, *J.C. de Moraes 1936* (RB00272388 – image!); **Paraná**: Londrina, 17 Nov 1969, *G. Hatschbach 22898* (NYBG00779051 – image!); **Pernambuco**: [without exact location] 1838, *Gardner 1138* (P04598076); Petrolina Mun., Tapera, Aug 1930, *D.B. Pickel 50* (B, BM000019250); **Rio de Janeiro**: Rio de Janeiro [city], 1883, *A. Glaziou 15355* (LE); **Roraima**: Rio Ajarani, 29 Apr 1974, J.*M. Pires et al. 14408* (IAN144038 – image!); **São Paulo**: Rio Negro, between Ilha Uabetuba & Ilha da Silva, 14 Oct 1971, *G.T. Prance et al. 15235* (G, K, M, P04598013, U1473419); **Sergipe**: Cristinapolis Mun., 2 Apr 1976, *G. Davidse et al. 11810* (U1473430); **Tocantins**: Darcinopolis Mun., Rio Tocantins, 16 Apr 2008, *G. Pereira-Silva et al. 12933* (CEN00091111 – image!); **COLOMBIA**: Rio Vaupés, 1852, *R. Spruce 2546* (E, K); **ECUADOR**: El Oro, 18 km on road Huaquillas–Arenillas, [without date] *Harling & Andersson 18835* (GB – image!); **GUIANA**: [without exact location] 1868, *Schomburgk 835* (B, E, K, P04598085); Pirara, 1841, *Schomburgk 325* (BM000019270); Region Upper Takutu, Essequibo, 1 Jun 1996, *D. Clarke 1874* (U1473431); **PARAGUAY**: **Amambay Dept**.: between Rio Apa & Rio Aquidabán, 13 Feb 1908, *K. Fiebrig 4928* (BM000019221, G, E, K, L); **Cordillera Dept**.: see lectotype of M.glochidiataf.lanceolata; Caraguatay, Aug 1900, *E. Hassler 3126* (B, BM000019232, G, P00743953, P00743951, P00743952); **San Pedro Dept**.: [without exact location] 26 Oct 1953, *A.L. Woolston 210* (K); **PERU**: **Cusco Region**: Paucartambo prov., Cusco dept., Pillcopata, 20 Jun 1959, *J. Infantes 5913* (B); **Puno Region**: Carabaya prov., San Gaban distr., [without date] ex herb. Steudel *2288* (G, P04598108); **SURINAME**: nr Paramaribo, 12 Feb 1904, *van Hell 150* (U1473435); Paramaribo, 10 April 1916, *J.A. Samuels 8* (K); Paramaribo, 12 Apr 1916, *J.A. Samuels 65* (BM000019265, L1678237, P04598014); Found as alien in Southeast Asia (Fig. 24): **INDONESIA**: Java, Pasuruan, 1924, *anonymous s.n.* (L1678238). Previously not reported from this region (Backer 1954).

##### 10. M. tenuifolia

Taxon classificationPlantaeCaryophyllalesMicroteaceae

Moq. in DC., Prodr. 13(2): 18 (1849).

M.maypurensisvar.angustifolia Moq. in DC., Prodr. 13(2): 18 (1849). Lectotype (Sennikov & Sukhorukov, designated here): BRAZIL. Jacobina Mountains in Bahia, 1836, Blanchet 2588 (P00798998!).

###### Lectotype.

(designated by Nowicke 1968: 352): BRAZIL. Espírito Santo State, Pico d’Habira [Pico do Itabira], 1843, *Claussen 392* (P00743993!).

###### Description.

Annual or short-lived perennial herb with several or numerous stems 10–40 cm high; leaves sessile (sometimes rosulate leaves shortly pedunculate), cuneate, 10–30 mm long and 0.3–3.0(5.0) mm wide; inflorescence a spike; pedicels 1.35–1.7(2.5) mm; flowers with a bract and two bracteoles, perianth segments 5, white, oblong; stamens 6–8, stigmas 3–5, thin; fruit 0.9–1.1 × 1.0–1.1 mm (Fig. 8E), equal to perianth or slightly protruding; pericarp smooth or with small and scattered tubercles (Fig. 8F), more or less reticulate, readily scraped off the seed; seed 0.9–1.1 mm, with rough surface (Fig. 8H).

We report for the first time that the perianth/fruit ratio is a useful distinguishing character for this species. Also, the number of stigmas can be useful in delimiting *M.tenuifolia* and similar forms of *M.celosioides* with narrower leaves. The character set of *M.tenuifolia* supports its close relationship to *M.maypurensis*, especially the forms with reduced pericarp outgrowths. Remarkably, Moquin-Tandon (1849) described a new variety of *M.maypurensis* (var. angustifolia Moq.) represented by two specimens of *M.tenuifolia* (P00743955! and P00798998!) and one specimen containing two individuals, *M.tenuifolia* and a narrow-leaved *M.maypurensis* (leg. *Blanchet 2588*, P00743954!). The figure of *M.tenuifolia* in Calió and Pirani (2006) showing the tuberculate perianth is rather an exception, and the individuals with pericarp lacking the outgrowths have so far been collected more frequently.

###### Habitat.

Forest margins, rocky places; 0–1000 m.

###### Distribution.

**Figure 25.**
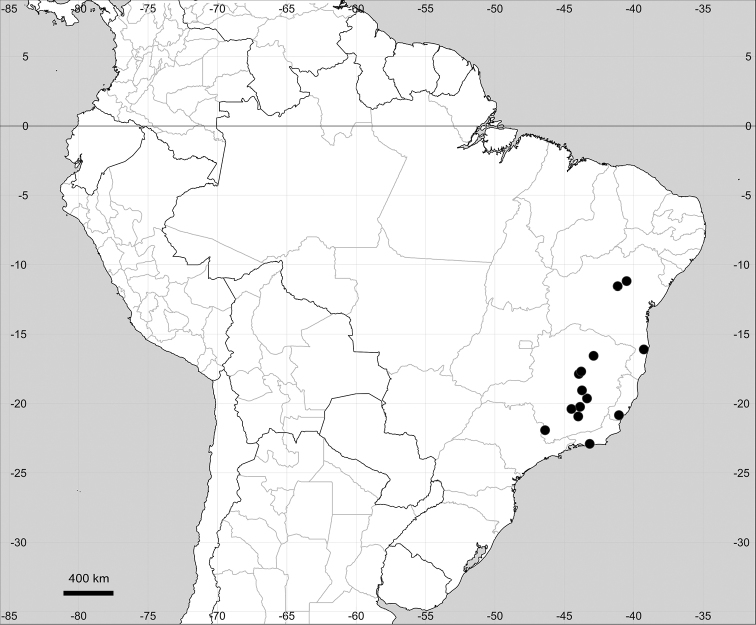
Distribution map of *Microteatenuifolia*.

Endemic to Eastern Brazil, found only in Bahia, Espírito Santo, Minas Gerais and Rio de Janeiro States (Fig. 25).

###### Specimens examined.

**BRAZIL**: **Bahia**: 4 km SW of Belmonte, 23 Mar 1974, *R.M. Harley 17305* (K, P04598081, U17305, U1473428); see lectotype of M.maypurensisvar.angustifolia; Jacobina, [without date], *Blanchet 153* (BM000019209); Morro do Chapeu, 1000 m, 1 Jun 1980, *R.M. Harley 22924* (U1473452); **Espírito Santo**: see lectotype of *M.tenuifolia*; **Minas Gerais**: [without exact location] Feb 1839, *Riedel 48* (P007990000); [without exact location] 1841, *Claussen 4* (P00743934); [without exact location] 1845, *Widgren s.n.* (M); Caldas, 5 May 1870, *A. Glaziou 11* (P04598116); Caldas, 15 May 1870, *A.T. Regnell s.n.* (B); Serrra das Vertentes, Jun 1893, *A. Glaziou 20437* (B, K, P04598119); Turvo, 24 Apr 1926, *W. Hohne & A. Gehrt s.n.* (PACA 76840); Buenópolis Mun., Curimatai, [without date] *A. Glaziou 13127* (P04598093); Buenópolis Mun., Curimatai, [without date] *A. Glaziou 19399* (P04598074); Serra do Espinhaco, 15 Feb 1969, *H.S. Irwin et al. 23315* (G); Bacia do Córrego Escurona, Grão Mogol, 2 Nov 1987, *M.C. Assis & al. s.n.* (PACA 76297); Vale do Rio Itacambiruçu, Grão Mogol, 10 Dec 1989, *A. Freire-Fierres et al. s.n.* (PACA 76298); Vale do Córrgo Escurona, Grão Mogol, 13 Jun 1990, *A.A. Oliveira et al. s.n.* (PACA 76299); Serra dos Inconfidentes, Pico do Itabirito, Itabirito, 4 Jan 1994, *W.A. Teixeira s.n.* (PACA); Estação Ecológica da Mata do Cedro, Carmópolis, 13 Jul 2004, *L. Echternacht & T. Dornas 573* (PACA); Estação Ecológica da Mata do Cedro, Carmópolis, 23 Jan 2005, *L. Echternacht & T. Dornas 830* (PACA); Grão Mogol Mun., Parque Estadual de Grão Mogol, 13 Apr 2006, *C.V. Vidal 177* (PACA); Santana do Riacho Mun., Entre a Rodovia BR 251 e Grão Mogol, Cachoeira Véu de Noiva, 15 Mar 2007, *M.S. Marchioretto 352 & 355* (PACA); **Rio de Janeiro**: nr Rio de Janeiro, Feb 1882, *A. Glaziou 13127* (G, K); Rio de Janeiro, 1887, *A. Glaziou 17748* (LE).

#### Species excluded

All species cited below under *Microtea* belong to the South African genus *Lophiocarpus* Turcz., with a distinct position within Caryophyllales (Cuénoud et al. 2002, Schäferhoff et al. 2009) and with a different seed anatomy (Sukhorukov et al. 2015). The transfers of *Lophiocarpus* to *Microtea* were undertaken due to the morphological similarity of their members (Brown 1909), which is a case of homoplasy between phylogenetically distant Caryophyllales genera (Schäferhoff et al. 2009, Brockington et al. 2009).

*Microteaburchellii* (Hook.f.) N.E.Br., Bull. Misc. Inf. Kew 1909(3): 135 [1909]

≡ ***Lophiocarpusburchelii*** Hook.f. in Bentham & Hooker f., Gen. Pl. 3(1): 50 (1880);

*Microteagracilis* A.W.Hill, Bull. Misc. Inf. Kew 1910(2): 56 [1910]

= ***Lophiocarpuspolystachyus*** Turcz., Bull. Soc. Imp. Naturalistes Moscou 16: 56 (1843);

*Microteapolystachya* (Turcz.) N.E.Br., Bull. Misc. Inf. Kew 1909(3): 135 [1909]

≡ ***Lophiocarpuspolystachyus*** Turcz., Bull. Soc. Imp. Naturalistes Moscou 16: 56 (1843);

*Microteatenuissima* (Hook.f.) N.E.Br., Bull. Misc. Inf. Kew 1909(3): 134 [1909]

≡ ***Lophiocarpustenuissimus*** Hook.f., Hooker’s Icon. Pl. 15: 50, tab. 1463 (1883).

## Supplementary Material

XML Treatment for Microtea

XML Treatment for Microtea subgen. Microtea

XML Treatment for M. debilis

XML Treatment for M. celosioides

XML Treatment for M. papillosa

XML Treatment for M. scabrida

XML Treatment for M. sulcicaulis

XML Treatment for M. bahiensis

XML Treatment for M. portoricensis

XML Treatment for Microtea subgen. Ancistrocarpus

XML Treatment for M. glochidiata

XML Treatment for M. maypurensis

XML Treatment for M. tenuifolia
